# High Levels of Multiple Phage WO Infections and Its Evolutionary Dynamics Associated With *Wolbachia*-Infected Butterflies

**DOI:** 10.3389/fmicb.2022.865227

**Published:** 2022-04-21

**Authors:** Shuo Gao, Ye-Song Ren, Cheng-Yuan Su, Dao-Hong Zhu

**Affiliations:** Laboratory of Insect Behavior and Evolutionary Ecology, College of Life Science and Technology, Central South University of Forestry and Technology (CSUFT), Changsha, China

**Keywords:** phage WO, multiple infections, recombination, horizontal transfer, *Wolbachia*, butterfly, Lepidoptera

## Abstract

*Wolbachia* is a maternally inherited bacterium that is widely distributed among arthropods, in which it manipulates the reproduction of its hosts. Phage WO is the only bacteriophage known to infect *Wolbachia*, and may provide benefit to its host or arthropods. We screened for the presence of phage WO in *Wolbachia*-infected butterfly species for the first time, to investigate their diversity and evolutionary dynamics. All *Wolbachia*-infected butterfly species, including members of the families Hesperiidae, Lycaenidae, Nymphalidae, Papilionidae, and Pieridae, were found to harbor phage WO. Interestingly, 84% of 19 butterfly species, which were infected with a single *Wolbachia* strain harbored high levels of multiple phage types (ranging from 3 to 17 types), another three species harbored one or two phage types. For *Wolbachia* strains (ST-41, ST-19, ST-125 and ST-374) shared among various butterfly species, their host insects all harbored multiple phage types, while two *Wolbachia* strains (ST-297 and ST-wPcau) were found to infect one butterfly species, whose insect hosts harbored a single phage type, suggesting that horizontal transfer of *Wolbachia* between insects increased the likelihood of exposure to phages, resulting in increased phage genetic diversity. Twelve horizontal transmission events of phage WO were found, which shared common phage WO types among different *Wolbachia* strains associated with butterflies. Most horizontal transfer events involved different *Wolbachia* supergroups (A and B). Horizontal acquisition of phage WO might also occur between eukaryotes without *Wolbachia* transfer. Furthermore, 22 putative recombination events were identified in 13 of 16 butterfly species which harbored multiple phage types. These results showed that horizontal transfer of *Wolbachia* caused it to be exposed to the phage gene pool, and that horizontal transmission of phage WO, as well as intragenic recombination were important dynamics for phage WO genome evolution, which effectively promoted the high level of phage WO diversity associated with butterflies.

## Introduction

*Wolbachia* (Anaplasmataceae) are maternally inherited endosymbiotic bacteria that infect arthropods and filarial nematodes (Werren et al., 2008; Engelstädter and Hurst, 2009). They are extremely widespread and probably occur in 40–65% of arthropod species (Hilgenboecker et al., 2008; Zug and Hammerstein, 2012; Weinert et al., 2015). Although vertical transmission of *Wolbachia* from mother to offspring predominates within species, horizontal transmission between species often occurs in arthropods (Sintupachee et al., 2006; Yang et al., 2013; Johannesen, 2017; Cooper et al., 2019; Hou et al., 2020; Sanaei et al., 2021). *Wolbachia* manipulates its host’s reproduction by inducing several phenotypes, such as cytoplasmic incompatibility (CI), parthenogenesis, feminization of genetic males, and male-killing (Werren et al., 2008).

Bacterial viruses (bacteriophages or phages) are the most abundant organisms on earth and constitute a significant force in bacterial genome evolution (Hendrix et al., 1999; Bordenstein and Wernegreen, 2004). As a consequence of reductive evolution, mobile DNA elements were thought to be rare or absent in obligate intracellular bacteria given their isolated niche, but increasing reports have shown that the ecology of bacterial endosymbionts significantly influenced the amount of their genome populated by mobile elements. Endosymbionts that are strictly vertically transmitted from parents to offspring often lack mobile genetic elements, while intracellular bacteria that may be horizontally transmitted often retain a large amount of mobile DNA, including phages (Moran and Plague, 2004; Bordenstein and Reznikoff, 2005; Newton and Bordenstein, 2011; Metcalf and Bordenstein, 2012). *Wolbachia* phage, a λ phage-like temperate dsDNA phage, named phage WO, was first characterized from the *Wolbachia* strain wTai, infecting *Teleogryllus taiwanemma*, and can be either lysogenic and integrated into the *Wolbachia* chromosome, or lytic and free in the cytoplasm (Masui et al., 2000, 2001). Phage WO is estimated to infect about 90% of supergroups A and B of *Wolbachia* from various arthropod groups, but is absent from the mutualistic C and D supergroups commonly found in filarial nematodes (Bordenstein and Wernegreen, 2004; Gavotte et al., 2007; Gerth et al., 2014). Nearly all sequenced *Wolbachia* genomes, except those acting as obligate mutualists, harbored phage WO (Gavotte et al., 2007; Kent and Bordenstein, 2010; Metcalf and Bordenstein, 2012). Moreover, *Wolbachia* not currently infected by phages often show evidence of past infections (Foster et al., 2005; Kent and Bordenstein, 2010). An *orf7*-like phage pseudogene has even been found in a newly described *Wolbachia*-free gall wasp species, *Latuspina jinzhaiensis*, suggesting that vestiges of prophage DNA remain in the chromosomes of the host insect after a previous lateral gene transfer event (Abe et al., 2021; Zhu et al., 2021). Additionally, most phage-infected *Wolbachia* strains display low numbers of phage types, with 85% showing only one or two different phage types (Gavotte et al., 2007; Tanaka et al., 2009). On the other hand, multiple phage infections, where a *Wolbachia* strain displays more than two phage types, have been reported in several *Wolbachia* strains (Chauvatcharin et al., 2006; Gavotte et al., 2007). The results of Zhu et al. (2021) confirmed that a gall wasp, *Andricus hakonensis*, harbored 27 phage WO types. Thus, considering the wide distribution of *Wolbachia*, phage WO may be one of the most abundant phage lineages in arthropods (Zhu et al., 2021).

The persistence of the phage despite its documented lytic activity has led to the hypothesis that phage WO provides benefit to its *Wolbachia* or arthropod host (Kent and Bordenstein, 2010). As in other prokaryotes, the integration and transformation of prophages are considered major sources of *Wolbachia* lateral gene acquisition (Bordenstein et al., 2006). Phage WO may mediate lateral gene transfer between *Wolbachia* strains (Ishmael et al., 2009; Wang G. H. et al., 2016) and regulate *Wolbachia* density by inhibiting their replication or inducing cell lysis (Bordenstein et al., 2006). Recently, the factors underlying CI were identified as two genes, *cifA* and *cifB*, located adjacent to one another within WO prophage regions in the *Wolbachia* genome, with homologs in all known CI-inducing *Wolbachia* strains (Beckmann et al., 2017; LePage et al., 2017; Chen et al., 2019; Shropshire and Bordenstein, 2019). Mutation, recombination, and genome segment reassortment during replication may mediate genetic changes in viruses (Domingo, 2010). A phage genome can be divided into functional units (modules; each one responsible for head or tail formation, lysis, lysogeny, and so forth). It is generally believed that mixing through fragment rearrangement with other phages in the common gene pool comprise the main evolutionary dynamics of dsDNA phages (Hatfull, 2008). However, although phage WO is modular, it does not evolve according to modular gene exchange, but rather through point mutation, intragenic recombination, deletion, and purifying selection, given its intracellular habitat (Kent et al., 2011). The nucleotide sequence of the minor capsid gene *orf7* from the wKueA1 strain of *Wolbachia*, infecting *Ephestia kuehniella*, is chimeric, and population genetic analysis has confirmed the occurrence of intragenic recombination events (Bordenstein and Wernegreen, 2004). Zhu et al. (2021) provided empirical molecular evidence of *orf7* gene recombination, suggesting that intragenic recombination was the important evolutionary force, which effectively promoted the diversity of phage WO associated with gall wasps which harbor multiple phage WO types. Furthermore, base deletions during replication also significantly promoted the evolution of the phage genome, resulting in the diversity of phage WO associated with *A. hakonensis* (Su et al., 2021).

The order Lepidoptera (moths and butterflies) contains about 158,000 described species and it is one of the most widespread and diverse insect orders (van Nieukerken et al., 2011). Given its economic and ecological importance, the order Lepidoptera has been investigated extensively by entomologists and ecologists. The relationships between Lepidoptera and their heritable endosymbionts have received increasing attention. *Wolbachia* infections have been detected in many Lepidoptera, and in diverse butterfly species from various regions. They were present in 16% of 43 Lepidoptera species tested in Panama (Werren et al., 1995), in 58% of 120 Lepidoptera species in West Siberia, in particular, in 59% of 108 butterfly species (Ilinsky and Kosterin, 2017), in 45% of 49 butterfly species in Japan (Tagami and Miura, 2004), in 50% of 56 butterfly species in India (Salunke et al., 2012), in 35% of 52 butterfly species in southern China (Zhu and Gao, 2021), and in 43% of 53 *Mylothris* spp. and in 46% of 21 *Bicyclus* spp. (Duplouy et al., 2020), and in 29% of 24 *Acraea* spp. (Jiggins et al., 2001) in Africa. Many *Wolbachia* strains that infect Lepidoptera have evolved the ability to alter host reproduction; key mechanisms are inducing CI or shifting the sex ratio toward female prevalence in populations by male killing or feminization (Jiggins et al., 2001; Narita et al., 2011; Duplouy and Hornett, 2018). Lepidoptera are considered one of model systems for studying the manipulation of host reproduction by endosymbionts (Duplouy and Hornett, 2018). Given its potential impact on host genome evolution and reproductive regulation in insect hosts infected by *Wolbachia*, phage WO has received heightened interest. Nevertheless, only a minimal part of the phage biodiversity harbored in *Wolbachia*-infected Lepidoptera has been described (Tanaka et al., 2009; Batista et al., 2010; Furukawa et al., 2012; Wang et al., 2020). In this study, the presence of phage WO in 19 *Wolbachia*-infected butterfly species collected from China was detected by employing a PCR-based method with phage WO-specific gene markers in order to understand the prevalence patterns. To explore the evolutionary dynamics of phage WO diversity, we also analyzed intragenic recombination using the recombination detection procedure (RDP5) package and investigated horizontal transmission by comparative phylogenetic analysis of phages and their hosts.

## Materials and Methods

### Insect Collection

Adult butterflies were collected from Liaoning, Hunan and Guangdong Provinces, China, from June 2019 to September 2020. In total, 148 individuals of 56 species were obtained from five families (Supplementary Table 1). The adult butterflies were taken back to the laboratory where their abdomens were removed with sterile scissors and stored in 100% ethanol at -80°C for subsequent DNA extraction.

### DNA Extraction, PCR and Sequencing

The penultimate abdominal segments of all collected butterfly individuals were used for DNA extraction. The samples were soaked and cleaned twice in ultrapure water before DNA extraction. Total DNA was extracted from each individual using SDS/proteinase K digestion and a phenol-chloroform extraction method, as described previously (Zhu et al., 2007). The quality of all the DNA extracts was confirmed by PCR amplification of the *COI* gene with universal insect primer pairs (Folmer et al., 1994). *Wolbachia* infection screening were performed according to the methods of Zhu and Gao (2021). *Wolbachia* multi-locus sequence type (MLST) genes (*gatB*, *coxA*, *hcpA*, *fbpA*, and *ftsZ*) were amplified in *Wolbachia*-positive samples using the respective primers reported by Baldo et al. (2006).

Phage WO infections were screened by PCR using the primers WO-F (5’-CCCACATGAGCCAATGACGTCTG-3’) and WO-R (5’- CGTTCGCTCTGCAAGTAACTCCATTAAAAC-3’) to amplify a portion of the capsid protein gene *orf7* (Masui et al., 2000). PCR amplification was conducted using a C1000 Touch thermal cycler (Bio-Rad, Hercules, CA, United States) in a 25-μL reaction volume comprising 2.5 μL 10 × PCR buffer, 0.125 μL *Taq* polymerase (Takara, Dalian, China), 2 μL dNTPs (10 mmol each), 1 μL forward and reverse primer (10 μM), 1 μL extracted DNA, and 18.4 μL ddH_2_O. The cycling conditions were 95°C for 3 min, 35 cycles of 95°C for 30 s, 57°C for 40 s, and 72°C for 40 s. All reactions were followed by a final extension step for 5 min at 72°C.

One to three individuals from each butterfly species were used to sequence the *orf7* fragment. The PCR products were subsequently purified using a TaKaRa MiniBEST Agarose Gel DNA Extraction Kit Ver. 4.0 (Takara Biomedical Technology Co.), and the *orf7* gene fragments were directly sequenced from purified PCR products using PCR primers. The appearance of multiple peaks in a sample at initial sequencing was taken as an indication of multiple infections. The PCR products were then purified using a DNA gene gel extraction kit and ligated directly into the vector, following the manufacturer’s protocols. For each sample, 10–20 independent positive colonies were isolated and cultured in a lysogeny broth medium fortified with ampicillin. Plasmids were extracted and partially sequenced in both directions using an ABI 3730XLDNA sequencer (Applied Biosystems, Foster City, CA, United States) with M13F/R at Wuhan Icongene Co., Ltd.

### Phage WO Typing and Phylogenetic Analysis

Sequence homology analysis was first performed using the BLAST^1^ program online. Genetic distances between all sequence pairs were calculated using the Kimura 2-parameter distance model in MEGA 7. Sequences having greater than 1.5% nucleotide diversity in the *orf7* gene were defined as different haplotypes (Chafee et al., 2010; Zhu et al., 2021). The sequences have been deposited in GenBank under the following accession numbers: OM472181– OM472424.

The sequences obtained for the phage WO *orf7* gene and *Wolbachia* multi-locus sequence typing (MLST) genes were aligned using SEQMAN PRO v.11.2 (DNASTAR, Madison, WI, United States). The data sets were then analyzed using a maximum likelihood (ML) method implemented in PAUP v.4.0b (Swofford, 2003). The best evolutionary model was selected according to the corrected Akaike information criterion, implemented in MEGA v.7.0. The ML trees were analyzed for 1,000 bootstrap replicates to assess branch support.

### Recombination Analysis

The individual segment alignments were analyzed using different methods described in the Recombination Detection Program (RDP5) package to detect evidence of intragenic recombination (Heath et al., 2006). The six recombination detection methods implemented in the RDP5 program for the identification of recombinant sequences and breakpoints were as follows: 3Seq (Martin and Rybicki, 2000), BootScan/rescan recombination test (Martin et al., 2005), GENECONV (Padidam et al., 1999), MaxChi (Smith, 1992), Chimaera (Posada and Crandall, 2001), and the Siscan method (Gibbs et al., 2000). The default settings were used for all methods, and the highest acceptable *P*-value cutoff was set to 0.05.

## Results

### Multiple Phage WO Infections Associated With Butterflies

We collected 56 butterfly species belonging to five families from China. Among these, 19 species were infected with *Wolbachia*; two species, *Limenitis doerriesi* Staudinger and *Parnassius stubbendorfii* Menetries, were found to be infected with *Wolbachia* in this study, and the other 17 species detected by Zhu and Gao (2021; Supplementary Table 1). Following the method previously described (Zhu and Gao, 2021), the complete MLST and WSP profiles of the newly found *Wolbachia* strains were identified, and one unique ST and two *wsp* alleles were obtained (Table 1). Using the diagnostic PCR approach with the phage minor capsid protein gene (*orf7*)-specific primers, *Wolbachia*-infected species were screened for phage WO infection. Phage WO was found to be harbored in all *Wolbachia*-infected butterfly species (Table 1).

**TABLE 1:** *Wolbachia* strains and phage WO types in butterflies.

Host species		*Wolbachia* strains			Phage infection	WO type number
Family	Species	ST^b^	*wsp* allele	Supergroup		
Hesperiidae	*Isoteinon* sp.	ST-41	wsp-10	B	+ (1)^c^	9 (1)^d^
	*Notocrypta* sp.	ST-41	wsp-10	B	+ (1)	8 (1)
	*Ochlodes thibetana*	ST-374	wsp-64	B	+ (1)	5 (1)
Lycaenidae	*Pseudozizeeria maha*	ST-41	wsp-10	B	+ (2)	17 (2)
Nymphalidae	*Ariadne ariadne*	ST-41	wsp-10	B	+ (1)	3 (1)
	*Junonia almana*	ST-125	wsp-10	B	+ (2)	8 (2)
	*Limenitis doerriesi* ^a^	ST-297	wsp-61	B	+ (3)	1 (3)
	*Mycalesis francisca*	–	wsp-10	B	+ (1)	9 (1)
	*Polygonia c-aureum*	ST-wPcau	wsp-266	B	+ (3)	1 (3)
	*Stichophthalma* sp.	ST-374	wsp-64	B	+ (1)	2 (1)
	*Vanessa indica*	ST-125	wsp-10	B	+ (1)	8 (1)
	*Ypthima praenubila*	ST-19	wsp-108	A	+ (1)	10 (1)
	*Ypthima* sp.	ST-19	wsp-108	A	+ (1)	6 (1)
Papilionidae	*Parnassius stubbendorfii^a^*	–	wsp-369	B	+ (9)	7 (3)
Pieridae	*Colias croceus*	ST-141	wsp-61	B	+ (2)	8 (2)
	*Delias agostina*	–	wsp-10	B	+ (1)	7 (1)
	*Eurema blanda*	ST-41	wsp-10	B	+ (8)	8 (3)
	*Eurema hecabe*	ST-41	wsp-10	B	+ (1)	5 (1)
	*Leptosia nina*	ST-41	wsp-10	B	+ (2)	4 (2)

*^a^The Wolbachia strains were identified in this study, and others by Zhu and Gao (2021).*

*^b^ST = Wolbachia multi-locus sequence type (MLST), – = no data obtained.*

*^c^The number in parentheses refers to the number of insect individuals used for screening, and all tested individuals harbored phage WO.*

*^d^The number in parentheses refers to the number of insect individuals used for orf7 sequencing.*

In total, 259 phage WO *orf7* sequences were gained from 19 butterfly species infected with *Wolbachia*. Among these, 15 *orf7*-like sequences contained abnormal stop codons without being transcribed, which were treated as pseudogenes (accession no.: OM663002– OM663016). They were excluded from subsequent analysis. Phage WO types with similarity of *orf7* DNA sequences greater than 98.5% were defined as identical types in accordance with a previous study (Chafee et al., 2010; Zhu et al., 2021). We named phage types as WO followed by the abbreviation of the insect name and haplotype numbers. *L. doerriesi* and *Polygonia c-aureum* L. were found to harbor a single phage type, WOLdo and WOPca, respectively, and *Stichophthalma* sp. harbored two phage types, WOSsp-1 and WOSsp-2. Unexpectedly, the other 16 *Wolbachia*-infected butterfly species (84% of 19 species tested) harbored multiple phage types: *Ypthima praenubila* Leech and *Pseudozizeeria maha* (Kollar) harbored 10 and 17 types, and the other species carried 3–9 types, respectively, while their *Wolbachia* infections were all of a single strain (Table 1 and Supplementary Figures 1–4).

### Phage WO Diversity Within *Wolbachia* Strains

**FIGURE 1:**
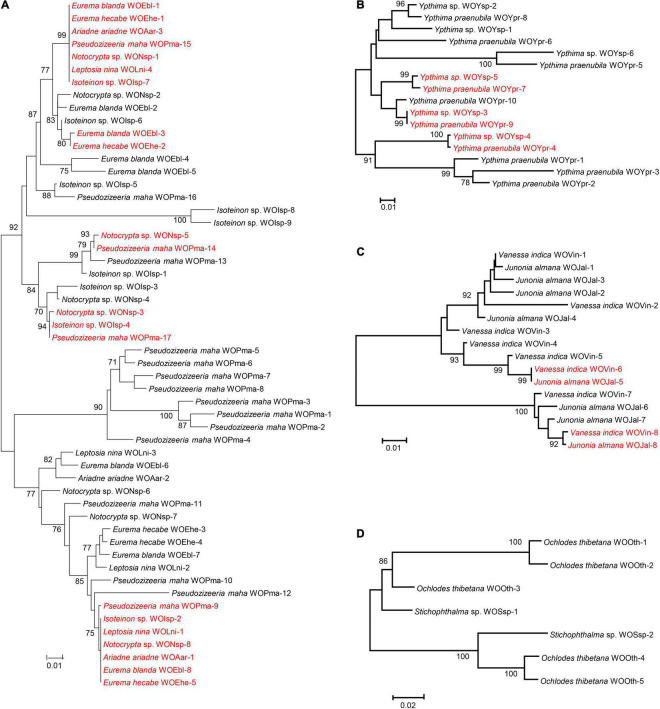
Maximum likelihood phylogenetic tree of the phage WO *orf7* nucleotide sequences from *Wolbachia* strains ST-41 **(A)**, ST-19 **(B)**, ST-125 **(C)** and ST-374 **(D)**. Numbers above branches are bootstrap values computed from 1,000 replications. WOEbl-1 refers to phage WO type. Red font indicates identical *orf7* sequences or those with similarity of *orf7* sequences greater than 98.5% among their respective accomplices.

As shown in Table 1, four *Wolbachia* strains, that is, ST-41, ST-19, ST-125 and ST-374, were shared among various butterfly species. To clarify the phage WO infections within these *Wolbachia* strains, based on the *orf7* sequences, phylogenetic trees were constructed of the phage WO types infecting these *Wolbachia* strains from various insects, respectively (Figure 1).

*Wolbachia* strain ST-41 was found in seven butterfly species from four families (Hesperiidae, Lycaenidae, Nymphalidae, and Pieridae). These butterfly species harbored two common phage WO types; sequences of WOEbl-1, WOEhe-1, WOAar-3, WOPma-15, WONsp-1, WOLni-4 and WOIsp-7 were identical, and WOPma-9, WOIsp-2, WOLni-1, WONsp-8, WOAar-1, WOEbl-8 and WOEhe-5 were identical *orf7* sequences or had one base substitution. Similarly, *Eurema blanda* (Boisduval) and *E. hecabe* (L.); *Notocrypta* sp. and *P. maha*; *Notocrypta* sp., *Isoteinon* sp. and *P. maha* harbored a common phage WO type, respectively. However, the remaining 33 phage WO types were carried by only one butterfly species (Figure 1A).

Two *Ypthima* species infected with *Wolbachia* strain ST-19 and *Vanessa indica* Herbst and *Junonia almana* (L.) infected with *Wolbachia* strain ST-125 harbored three and two common phage WO types, respectively. Meanwhile, these insects all carried their own unique phage WO types (Figures 1B,C). However, no common phage WO types were detected between *Ochlodes thibetana* Oberthür and *Stichophthalma* sp., although they were infected with the same *Wolbachia* strain (ST-374; Figure 1D).

### Horizontal Transfer

**FIGURE 2:**
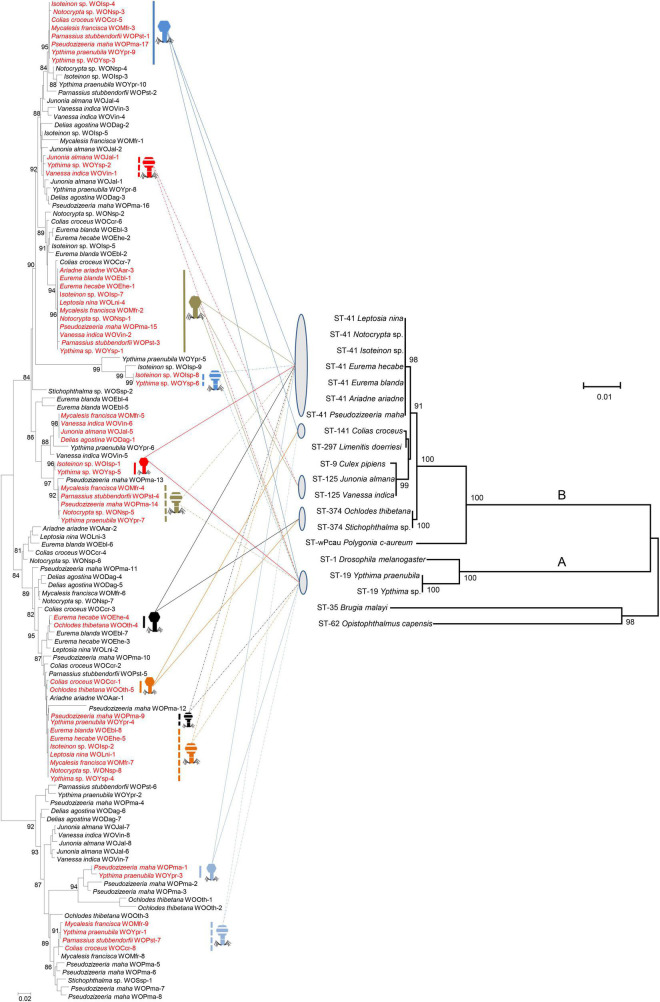
Comparison among phylogenies of phage WO based on *orf7* nucleotide sequences (left) and *Wolbachia* based on concatenated sequences of multi-locus sequence type (MLST) genes (right). Numbers above branches are bootstrap values computed from 1,000 replications. Red font indicates identical *orf7* sequences or those with similarity of *orf7* sequences greater than 98.5% among their respective accomplices. These accomplices are shown with icons of different colors and shapes. The capital letters on the right indicate the *Wolbachia* supergroups.

Phylogenetic reconstruction of phage WO *orf7* sequences and concatenated sequences of five *Wolbachia* MLST genes from butterflies was performed using ML methods (Figure 2). Obviously, there was no congruence between phage WO and its host *Wolbachia* phylogenies. Moreover, direct evidence was found for 12 horizontal transmission events of phage WO from butterflies, two involving three *Wolbachia* strains and 10 involving two *Wolbachia* strains, which shared common phage WO types among different *Wolbachia* strains. For example, WOIsp-4 (from *Isoteinon* sp.), WONsp-3 (from *Notocrypta* sp.), WOCcr-5 [from *Colias croceus* (Fourcroy)], WOMfr-3 (from *Mycalesis francisca* Cramer), WOPst-1 (from *P. stubbendorfii*), WOPma-17 (from *P. maha*), WOYpr-9 (from *Y. praenubila*), and WOYsp-3 (from *Ypthima* sp.) had identical *orf7* sequences or one base substitution, thus belonging to the same phage WO type. They infected at least three *Wolbachia* strains, namely ST-41, ST-141, and ST-19. The *Wolbachia* strains of *M. francisca* and *P. stubbendorfii* could not be characterized because of failure to amplify some MLST loci. Among 12 horizontal transmission events, 10 involved different *Wolbachia* supergroups, occurring between supergroup A and supergroup B.

### Intragenic Recombination

Multiple phage types are carried by one butterfly species with a single *Wolbachia* strain infection, therefore genetic material might be exchanged for reassortment and intragenic recombination. To obtain direct evidence of intragenic recombination, we performed recombination analysis of the aligned *orf7* sequences obtained from the same butterfly species using RDP5 programs. Regarding the results of recombination analysis, this study only provided the recombination events detected by more than three detection methods implemented in RDP5 programs.

**FIGURE 3:**
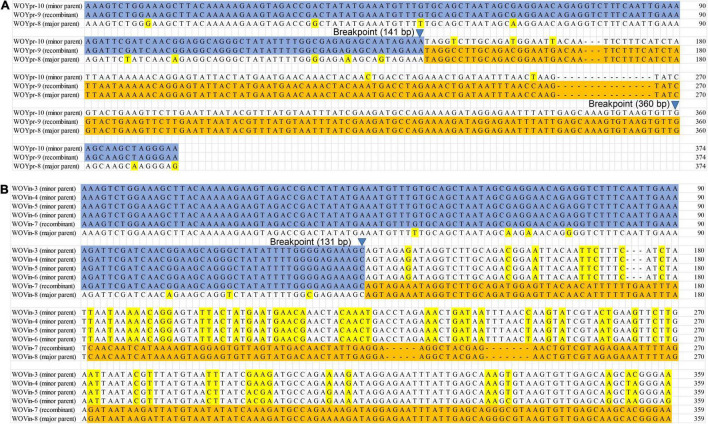
Recombination events of the *orf7* gene between WOYpr-8 and WOYpr-10 resulting in recombinant WOYpr-9 **(A)** and among WOVin-8 and WOVin-3, WOVin-4, WOVin-5, and WOVin-6 resulting in recombinant WOVin-7 **(B)**.

A total of 22 putative recombination events were identified, resulting in new phage types, from 13 to 16 butterfly species which harbored diverse phage WOs (Table 2, Figure 3 and Supplementary Figures 5–11). These recombination events include two types; one major parent and one minor parent or one major parent and two to four minor parents were recombined with the same breakpoint into a new phage WO lineage (Table 2). For example, Phage WO type WOYpr-9 from *Y. praenubila* was detected as a recombinant by four of the six methods used: 3Seq (*P* < 10^–9^), BootScan (*P* < 10^–8^), GENECONV (*P* < 10^–5^), and MaxChi (*P* < 10^–8^). The major and minor parents were WOYpr-8 and WOYpr-10, and the beginning breakpoints were 141 and 360 bp (Figure 3A). Type WOVin-7 from *V. indica* was detected as a recombinant by three of the six methods used: 3Seq (*P* < 10^–7^), BootScan (*P* < 10^–5^–10^–6^), and GENECONV (*P* < 10^–4^–10^–5^). The major parent was WOVin-8, and minor parents may be WOVin-3 or WOVin-4, WOVin-5, or WOVin-6; the beginning breakpoint was 131 bp (Figure 3B). Furthermore, WOPma-2, WOVin-4, WOYpr-9, WOYpr-10, WOPst-1, WOpst-2, WOCcr-3, and WOCcr-4, which were obtained by recombination, could also be used as parents for recombination to contribute to the diversity of phages (Table 2). These results suggested that intragenic recombination occurred widely and frequently in phage WO harbored in butterflies.

**TABLE 2:** Recombination analysis of the phage WO *orf7* gene in butterflies using six methods implemented in the RDP package.

Insect	Recombinant	Major parent	Minor parent	Breakpoint	Method	*P*-value
*Isoteinon* sp.						
	WOIsp-9	WOIsp-8	WOIsp-7 WOIsp-6	180	3Seq BootScan GENECONV MaxChi	7.69E-10 3.65E-07 5.68E-06 7.32 E-07
	WOIsp-4	WOIsp-1	WOIsp-5	180	3Seq BootScan GENECONV	3.78E-04 5.08E-04 6.35 E-05
*Notocrypta* sp.						
	WONsp-6	WONsp-7	WONsp-3 WONsp-4	263	3Seq BootScan GENECONV MaxChi	6.94E-08 6.47E-05 6.65E-08 4.36 E-07
*Ochlodes thibetana*						
	WOOth-4	WOOth-5	WOOth-3	90	3Seq BootScan GENECONV MaxChi	4.36E-06 2.36E-04 4.21E-07 1.27E-08
*Pseudozizeeria maha*						
	WOPma-2	WOPma-1	WOPma-8	263	3Seq BootScan GENECONV MaxChi	2.86E-08 6.33E-07 7.92E-07 6.44E-08
	WOPma-6	WOPma-8	WOPma-15	360	3Seq BooScan GENECONV MaxChi	5.434E-08 9.23E-05 4.56E-07 5.36 E-07
	WOPma-12	WOPma-9	WOPma-1 WOPma-2 WOPma-3	120	3Seq BootScan GENECONV MaxChi	4.32E-10 ∼ 9.36E-09 3.65E-09 ∼ 2.36E-09 1.23E-09 ∼ 8.21E-08 6.96E-08 ∼ 1.27E-07
*Vanessa indica*						
	WOVin-7	WOVin-8	WOVin-3 WOVin-4 WOVin-5 WOVin-6	131	3Seq BootScan GENECONV	8.65E-08 ∼ 4.21E-08 4.96E-06 ∼ 5.87E-07 3.24E-05 ∼ 3.21E-06
	WOVin-4	WOVin-5	WOVin-1	270	3Seq BootScan GENECONV MaxChi	3.87E-05 5.21E-04 4.98E-06 7.32E-05
*Ypthima praenubila*						
	WOYpr-2	WOYpr-3	WOYpr-1	180	3Seq BootScan GENECONV MaxChi	8.29E-09 4.69E-08 3.14E-07 4.72E-09
	WOYpr-9	WOYpr-8	WOYpr-10	141/360	3Seq BootScan GENECONV MaxChi	6.89E-10 4.29E-09 8.24E-06 1.92E-09
	WOYpr-10	WOYpr-7	WOYpr-9	270	3Seq BootScan GENECONV MaxChi	9.69E-10 5.79E-08 9.44E-07 4.62E-08
*Ypthima* sp.						
	WOYsp-3	WOYsp-5	WOYsp-2	218	3Seq BootScan GENECONV MaxChi	9.43E-09 6.23E-08 3.56E-07 5.36 E-08
*Parnassius stubbendorfii*						
	WOPst-2	WOPst-1	WOPst-7	117	3Seq BootScan GENECONV MaxChi	9.43E-09 6.23E-08 3.56E-07 5.36 E-08
	WOPst-1	WOPst-2	WOPst-4	117	3Seq BootScan GENECONV MaxChi	8.97E-06 6.35E-07 4.66E-07 1.36 E-09
*Colias croceus*						
	WOCcr-4	WOCcr-3	WOCcr-6	290	3Seq BootScan GENECONV MaxChi	4.96E-08 8.65E-07 7.56E-06 5.49E-08
	WOCcr-3	WOCcr-4	WOCcr-1	270	3Seq BootScan GENECONV MaxChi	7.62E-09 8.21E-06 9.26E-06 5.86E-08
*Delias agostina*						
	WODag-7	WODag-6	WODag-2	90	3Seq BootScan GENECONV	3.43E-09 8.28E-08 6.32E-07
	WODag-4	WODag-5	WODag-3	90	3Seq BootScan GENECONV	6.89E-09 6.23E-08 4.42E-07
*Eurema blanda*						
	WOEbl-3	WOEbl-1	WOEbl-8	131	3Seq GENECONV MaxChi	4.59E-09 6.32E-08 7.38E-07
*Eurema hecabe*						
	WOEhe-2	WOEhe-1	WOEhe-5	117	3Seq BootScan GENECONV MaxChi	3.55E-10 8.33E-07 5.64E-08 9.28E-09
*Leptosia nina*						
	WOLni-3	WOLni-1	WOLni-4	263	3Seq GENECONV MaxChi	6.96E-07 8.03E-08 7.08E-07

## Discussion

The prevalence of *Wolbachia* has been widely reported, and the bacterium has a high infection rate in butterflies (Jiggins et al., 2001; Tagami and Miura, 2004; Salunke et al., 2012; Ilinsky and Kosterin, 2017; Duplouy et al., 2020; Zhu and Gao, 2021). However, little information is available about phage WO associated with *Wolbachia-*infected butterflies. In this study, we detected the presence of phage WO infections associated with butterflies for the first time, and demonstrated that all 19 *Wolbachia*-infected species, belonging to Hesperiidae, Lycaenidae, Nymphalidae, Papilionidae, and Pieridae, harbor phage WO. Although multiple phage WO infections have been reported in several *Wolbachia* strains (Chauvatcharin et al., 2006; Gavotte et al., 2007), most phage-infected *Wolbachia* strains display low numbers of phage types, with 85% showing only one or two different phage types (Gavotte et al., 2007; Tanaka et al., 2009). Zhu et al. (2021) found that six gall wasp species with double or multiple *Wolbachia* infections harbored a high level of phage WO diversity. However, in that system, the number of phage types within a single *Wolbachia* strain remains unclear. Interestingly, in this study, the results indicated that 84% of 19 butterfly species which were infected with a single *Wolbachia* strain harbored high levels of multiple phage types: *Y. praenubila* and *P. maha* harbored 10 and 17 types, and the other 14 species carried 3–9 types, respectively. Although the number of positive clones selected was increased to 20 for samples with more than three types, the diversity of phage WO might still be underestimated in this study. The *Wolbachia* strain *w*Ri, infecting *Drosophila simulans*, showed a single WO haplotype according to the initial PCR detection using phage WO-specific gene markers, but the genome sequence confirmed four protophage copies in the genome, three of which were unique (Gavotte et al., 2007; Klasson et al., 2009). In fact, primers used to screen for the presence or absence of the *orf7* gene were not degraded enough to detect all *orf7* variants (Kent and Bordenstein, 2010; Su et al., 2021). Su et al. (2021) designed specific detection primers, and may have significantly improved the detection efficiency of *orf7* variants. Thus, the diversity of phage WO in *Wolbachia*-infected arthropods might be greater than current estimates.

The *Wolbachia* strains characterized in butterflies are often shared among various insect species, families and even orders (Ahmed et al., 2016; Ilinsky and Kosterin, 2017; Duplouy et al., 2020; Zhu and Gao, 2021). For example, strain ST-41, a core *Wolbachia* strain in butterflies, was shared among 30 butterfly species from four families, and it has also been found in four moth species from four families and one Diptera species (Ahmed et al., 2016; Ilinsky and Kosterin, 2017; Zhu and Gao, 2021). The occurrence of the similar *Wolbachia* strains in different butterfly species suggests that they may shift between host species via horizontal transfer, although the transfer mechanism is still not completely clear (Ahmed et al., 2016; Duplouy et al., 2020; Zhu and Gao, 2021). Horizontal transmission can increase the exposure of intracellular bacteria to the phage gene pool, thereby increasing the probability of being infected by phages (Moran and Plague, 2004; Bordenstein and Reznikoff, 2005; Newton and Bordenstein, 2011; Metcalf and Bordenstein, 2012). In this study, *Wolbachia* strains ST-41, ST-19, ST-125 and ST-374 were shared among two to seven butterfly species, respectively, and their host insects all harbored multiple phage types. In contrast, ST-297 and ST-wPcau were found to infect one butterfly species, whose insect hosts harbored a single phage type. Therefore, it might be considered that the horizontal transfer of *Wolbachia* between insect hosts increases the likelihood of exposure to phages, which is one of the reasons why butterflies harbor multiple types of phage WO.

Phage WO spreads among hosts through both vertical and horizontal transmission. Horizontal transfer of phage WO can occur between different *Wolbachia* strains, or it can be transmitted paternally by sperm from an infected male to the egg of a female carrying a phage-free *Wolbachia* (Gavotte et al., 2004; Bordenstein et al., 2006). The absence of an evolutionary correlation between WO and *Wolbachia* phylogenies (Bordenstein and Wernegreen, 2004; Gavotte et al., 2004; Wang N. X. et al., 2016) and divergent *Wolbachia* strains that infect the same (Gavotte et al., 2004; Chauvatcharin et al., 2006) or different hosts (Wang N. X. et al., 2016; Zhu et al., 2021) that have identical *orf7* sequences indicated that many horizontal phage WO transfers occurred between different *Wolbachia* endosymbionts. In this study, 12 horizontal transmission events of phage WO were found, which shared common phage WO types among different *Wolbachia* strains associated with butterflies. Most horizontal transfer events involved different *Wolbachia* supergroups (A and B). Prophages undergo a lytic phase capable of rupturing bacterial and eukaryotic cell membranes, and phage WO occurs in the extracellular matrix of arthropods. Thus, they might pass through the eukaryote cell wall and then initiate new infections (Masui et al., 2001; Bordenstein et al., 2006; Gavotte et al., 2007). *Wolbachia* strains ST-41, ST-19, ST-125 and ST-374 were shared among various butterfly species. In addition to the common types, these butterfly species all harbored a variety of species-specific phage WO types. These results strongly suggested that *Wolbachia* may obtain phage WO types in butterflies through horizontal transfer of virus between eukaryotes, without *Wolbachia*. Therefore, the horizontal transmission of phage WO, with or without *Wolbachia*, was an important route for butterfly infecting *Wolbachia* strains to obtain phage diversity.

Reassortment and recombination have been confirmed in RNA and DNA viruses as a mechanism to adapt to the changing environment, expand host range and increase virulence, resulting in the genetic diversity of viruses (Domingo, 2010; Franzo et al., 2016; Chen et al., 2018). Bordenstein and Wernegreen (2004) confirmed the recombinogenic nature of phage WO, and, in the case of the capsid protein gene *orf7*, the recombination rate was the fastest reported for the *Wolbachia* genome. Zhu et al. (2021) provided practical molecular evidence supporting *orf7* gene recombination in phage WO associated with gall wasps. Butterflies infected with a single *Wolbachia* strain carrying high levels of multiple phages provide an ideal model for detecting intragenic recombination. Twenty-two recombination events were identified in 13 of 16 butterfly species, which harbored diverse phage WO types, and some phage WO lineages derived from recombination could also be used as parents to form new phage WO types, displaying frequent and active recombination. These results suggested that intragenic recombination is an important evolutionary force, which effectively promotes the diversity of phage WO associated with butterflies.

## Data Availability Statement

The datasets presented in this study can be found in online repositories. The names of the repository/repositories and accession number(s) can be found below: https://www.ncbi.nlm.nih.gov/; OM472181 – OM472424, and OM663002–OM663016.

## Author Contributions

D-HZ and SG designed the study. SG, Y-SR, and C-YS performed experiments and analyses. D-HZ and Y-SR wrote the manuscript. All authors contributed to the article and approved the submitted version.

## Conflict of Interest

The authors declare that the research was conducted in the absence of any commercial or financial relationships that could be construed as a potential conflict of interest.

## Publisher’s Note

All claims expressed in this article are solely those of the authors and do not necessarily represent those of their affiliated organizations, or those of the publisher, the editors and the reviewers. Any product that may be evaluated in this article, or claim that may be made by its manufacturer, is not guaranteed or endorsed by the publisher.

## Footnotes

1
https://blast.ncbi.nlm.nih.gov/Blast.cgi

## References

[B1] Abe Y. Ide T. Su C. Y. Zhu D. H. (2021). Leaf galls with the same morphology induced on the same plant species by two species of *Latuspina* (Hymenoptera: Cynipidae), with a description of a new species. *Proc. Entomol. Soc. Wash.* 123 465–473. 10.4289/0013-8797.123.3.465

[B2] Ahmed M. Z. Breinholt J. W. Kawahara A. Y. (2016). Evidence for common horizontal transmission of *Wolbachia* among butterflies and moths. *BMC Evol. Biol.* 16:118. 10.1186/s12862-016-0660-x 27233666 PMC4882834

[B3] Baldo L. Bordenstein S. Wernegreen J. J. Werren J. H. (2006). Widespread recombination throughout *Wolbachia genomes*. *Mol. Bio. Evol.* 23 437–449. 10.1093/molbev/msj049 16267140

[B4] Batista P. D. Keddie B. A. Dosdall L. M. Harris H. L. (2010). Phylogenetic placement and evidence for horizontal transfer of *Wolbachia* in *Plutella xylostella* (Lepidoptera: Plutellidae) and its parasitoid, *Diadegma insulare* (Hymenoptera: Ichneumonidae). *Can. Entomol.* 142 57–64. 10.4039/n09-050

[B5] Beckmann J. F. Ronau J. A. Hochstrasser M. (2017). A *Wolbachia* deubiquitylating enzyme induces cytoplasmic incompatibility. *Nat. Microbiol.* 2:17007. 10.1038/nmicrobiol.2017.7 28248294 PMC5336136

[B6] Bordenstein S. R. Marshall M. L. Fry A. J. Kim U. Wernegreen J. J. (2006). The tripartite associations between bacteriophage, *Wolbachia*, and arthropods. *PLoS Pathog.* 2:e43. 10.1371/journal.ppat.0020043 16710453 PMC1463016

[B7] Bordenstein S. R. Reznikoff W. S. (2005). Mobile DNA in obligate intracellular bacteria. *Nat. Rev. Microbiol.* 3 688–699. 10.1038/nrmicro1233 16138097

[B8] Bordenstein S. R. Wernegreen J. J. (2004). Bacteriophage flux in endosymbionts (*Wolbachia*): infection frequency, lateral transfer, and recombination rates. *Mol. Biol. Evol.* 21 1981–1991. 10.1093/molbev/msh211 15254259

[B9] Chafee M. E. Funk D. J. Harrison R. G. Bordenstein S. R. (2010). Lateral phage transfer in obligate intracellular bacteria (*Wolbachia*): verification from natural populations. *Mol. Biol. Evol.* 27 501–505. 10.1093/molbev/msp275 19906794 PMC2822291

[B10] Chauvatcharin N. Ahantarig A. Baimai V. Kittayapong P. (2006). Bacteriophage WO-B and *Wolbachia* in natural mosquito hosts: infection incidence, transmission mode and relative density. *Mol. Ecol.* 15 2451–2461. 10.1111/j.1365-294X.2006.02947.x 16842419

[B11] Chen H. Ronau J. A. Beckmann J. Hochstrasser M. (2019). A *Wolbachia* nuclease and its binding partner provide a distinct mechanism for cytoplasmic incompatibility. *Proc. Natl. Acad. Sci. U.S.A.* 116 22314–22321. 10.1073/pnas.1914571116 31615889 PMC6825299

[B12] Chen Y. Trovao N. S. Wang G. Zhao W. He P. Zhou H. (2018). Emergence and evolution of novel Reassortant influenza a viruses in canines in southern China. *mBio* 9:e00909–18. 10.1128/mBio.00909-18 29871917 PMC5989073

[B13] Cooper B. S. Vanderpool D. Conner W. R. Matute D. R. Turelli M. (2019). *Wolbachia* acquisition by *Drosophila yakuba*-clade hosts and transfer of incompatibility loci between distantly related *Wolbachia*. *Genetics* 212 1399–1419. 10.1534/genetics.119.302349 31227544 PMC6707468

[B14] Domingo E. (2010). Mechanisms of viral emergence. *Vet. Res.* 41:38. 10.1051/vetres/2010010 20167200 PMC2831534

[B15] Duplouy A. Hornett E. A. (2018). Uncovering the hidden players in Lepidoptera biology: the heritable microbial endosymbionts. *PeerJ* 6:e4629. 10.7287/peerj.preprints.26768v1 29761037 PMC5947162

[B16] Duplouy A. Pranter R. Warren-Gash H. Tropek R. Wahlberg N. (2020). Towards unravelling *Wolbachia* global exchange: a contribution from the *Bicyclus* and *Mylothris* butterflies in the Afrotropics. *BMC Microbiol.* 20:319. 10.1186/s12866-020-02011-2 33081703 PMC7576836

[B17] Engelstädter J. Hurst G. D. D. (2009). The ecology and evolution of microbes that manipulate host reproduction. *Annu. Rev. Ecol. Evol. Syst.* 40 127–149. 10.1146/annurev.ecolsys.110308.120206

[B18] Folmer O. Black M. Hoeh W. Lutz R. Vrijenoek R. (1994). DNA primers for amplification of mitochondrial cytochrome c oxidase subunit I from diverse metazoan invertebrates. *Mol. Mar. Biol. Biotech.* 3 294–299. 7881515

[B19] Foster J. Ganatra M. Kamal I. Ware J. Makarova K. Ivanova N. (2005). The *Wolbachia* genome of *Brugia malayi*: endosymbiont evolution within a human pathogenic nematode. *PLoS Biol.* 3:e121. 10.1371/journal.pbio.0030121 15780005 PMC1069646

[B20] Franzo G. Cortey M. Segalés J. Hughes J. Drigo M. (2016). Phylodynamic analysis of porcine circovirus type 2 reveals global waves of emerging genotypes and the circulation of recombinant forms. *Mol. Phylogenet. Evol.* 100 269–280. 10.1016/j.ympev.2016.04.028 27114187

[B21] Furukawa S. Tanaka K. Ikeda T. Fukatsu T. Sasaki T. (2012). Quantitative analysis of the lytic cycle of WO phages infecting *Wolbachia*. *Appl. Entomol. Zool.* 47 449–456. 10.1007/s13355-012-0142-6

[B22] Gavotte L. Henri H. Stouthamer R. Charif D. Charlat S. Boulétreau M. (2007). A survey of the bacteriophage WO in the endosymbiotic bacteria *Wolbachia*. *Mol. Biol. Evol.* 24 427–435. 10.1093/molbev/msl171 17095536

[B23] Gavotte L. Vavre F. Henri H. Ravallec M. Stouthamer R. Boulétreau M. (2004). Diversity, distribution and specificity of WO phage infection in *Wolbachia* of four insect species. *Insect Mol. Biol.* 13 147–153. 10.1111/j.0962-1075.2004.00471.x 15056362

[B24] Gerth M. Gansauge M. T. Weigert A. Bleidorn C. (2014). Phylogenomic analyses uncover origin and spread of the *Wolbachia* pandemic. *Nat. Commun.* 5:5117. 10.1038/ncomms6117 25283608

[B25] Gibbs M. J. Armstrong J. S. Gibbs A. J. (2000). Sister-Scanning: a monte carlo procedure for assessing signals in recombinant sequences. *Bioinformatics* 16 573–582. 10.1093/bioinformatics/16.7.573 11038328

[B26] Hatfull G. F. (2008). Bacteriophage genomics. *Curr. Opin. Microbiol.* 11 447–453. 10.1016/j.mib.2008.09.004 18824125 PMC2706577

[B27] Heath L. Van Walt E. Varsani A. Martin D. P. (2006). Recombination patterns in aphthoviruses mirror those found in other picornaviruses. *J. Virol.* 80 11827–11832. 10.1128/JVI.01100-06 16971423 PMC1642601

[B28] Hendrix R. W. Smith M. C. Burns R. N. Ford M. E. Hatfull G. F. (1999). Evolutionary relationships among diverse bacteriophages and prophages: all the world’s a phage. *Proc. Natl. Acad. Sci. U.S.A.* 96 2192–2197. 10.1073/pnas.96.5.2192 10051617 PMC26759

[B29] Hilgenboecker K. Hammerstein P. Schlattmann P. Telschow A. Werren J. H. (2008). How many species are infected with *Wolbachia*? A statistical analysis of current data. *FEMS Microbiol. Lett.* 281 215–220. 10.1111/j.1574-6968.2008.01110.x 18312577 PMC2327208

[B30] Hou H. Q. Zhao G. Z. Su C. Y. Zhu D. H. (2020). *Wolbachia* prevalence patterns: horizontal transmission, recombination, and multiple infections in chestnut gall wasp-parasitoid communities. *Entomol. Exp. Appl.* 168 752–765. 10.1111/eea.12962

[B31] Ilinsky Y. Kosterin O. (2017). Molecular diversity of *Wolbachia* in Lepidoptera: prevalent allelic content and high recombination of MLST genes. *Mol. Phylogenet. Evol.* 109 164–179. 10.1016/j.ympev.2016.12.034 28082006

[B32] Ishmael N. Hotopp J. C. D. Ioannidis P. Biber S. Sakomoto J. Siozios S. (2009). Extensive genomic diversity of closely related *Wolbachia* strains. *Microbiology* 155 2211–2222. 10.1099/mic.0.027581-0 19389774 PMC2888116

[B33] Jiggins F. M. Bentley J. K. Majerus M. E. N. Hurst G. D. D. (2001). How many species are infected with *Wolbachia*? Cryptic sex ratio distorters revealed to be common by intensive sampling. *Proc. R. Soc. Lond. B* 268 1123–1126. 10.1098/rspb.2001.1632 11375098 PMC1088716

[B34] Johannesen J. (2017). Tracing the history and ecological context of *Wolbachia* double infection in a specialist host (*Urophora cardui*) – parasitoid (*Eurytoma serratulae*) system. *Ecol. Evol.* 7 986–996. 10.1002/ece3.2713 28168034 PMC5288247

[B35] Kent B. N. Bordenstein S. R. (2010). Phage WO of *Wolbachia*: lambda of the endosymbiont world. *Trends Microbiol.* 18 173–181. 10.1016/j.tim.2009.12.011 20083406 PMC2862486

[B36] Kent B. N. Funkhouser L. J. Setia S. Bordenstein S. R. (2011). Evolutionary genomics of a temperate bacteriophage in an obligate intracellular bacteria (*Wolbachia*). *PLoS One* 6:e24984. 10.1371/journal.pone.0024984 21949820 PMC3173496

[B37] Klasson L. Westberg J. Sapountzis P. Näslunda K. Lutnaes Y. Darby A. C. (2009). The mosaic genome structure of the *Wolbachia* wRi strain infecting *Drosophila simulans*. *Proc. Natl. Acad. Sci. U.S.A.* 106 5725–5730. 10.1073/pnas.0810753106 19307581 PMC2659715

[B38] LePage D. P. Metcalf J. A. Bordenstein S. R. On J. Perlmutter J. I. Shropshire J. D. (2017). Prophage WO genes recapitulate and enhance *Wolbachia*-induced cytoplasmic incompatibility. *Nature* 543 243–247. 10.1038/nature21391 28241146 PMC5358093

[B39] Martin D. Rybicki E. (2000). RDP: detection of recombination amongst aligned sequences. *Bioinformatics* 16 562–563. 10.1093/bioinformatics/16.6.562 10980155

[B40] Martin D. P. Posada D. Crandall K. A. Williamson C. (2005). A modified BOOTSCAN algorithm for automated identification of recombinant sequences and recombination breakpoints. *AIDS Res. Hum. Retrov.* 21 98–102. 10.1089/aid.2005.21.98 15665649

[B41] Masui S. Kamoda S. Sasaki T. Ishikawa H. (2000). Distribution and evolution of bacteriophage WO in *Wolbachia*, the endosymbiont causing sexual alterations in arthropods. *J. Mol. Evol.* 51 491–497. 10.1007/s002390010112 11080372

[B42] Masui S. Kuroiwa H. Sasaki T. Inui M. Kuroiwa T. Ishikawa H. (2001). Bacteriophage WO and virus-like particles in *Wolbachia*, an endosymbiont of arthropods. *Biochem. Biophys. Res. Commun.* 283 1099–1104. 10.1006/bbrc.2001.4906 11355885

[B43] Metcalf J. A. Bordenstein S. R. (2012). The complexity of virus systems: the case of endosymbionts. *Curr. Opin. Microbiol.* 15 546–552. 10.1016/j.mib.2012.04.010 22609369 PMC3424318

[B44] Moran N. A. Plague G. R. (2004). Genomic changes following host restriction in bacteria. *Curr. Opin. Genet. Dev.* 14 627–633. 10.1016/j.gde.2004.09.003 15531157

[B45] Narita S. Kageyama D. Hiroki M. Sanpei T. Hashimoto S. Kamito T. (2011). *Wolbachia*-induced feminisation newly found in *Eurema hecabe*, a sibling species of *Eurema mandarina* (Lepidoptera: Pieridae). *Ecol. Entomol.* 36 309–317. 10.1111/j.1365-2311.2011.01274.x

[B46] Newton I. L. G. Bordenstein S. R. (2011). Correlations between bacterial ecology and mobile DNA. *Curr. Microbiol.* 62 198–208. 10.1007/s00284-010-9693-3 20577742 PMC3006647

[B47] Padidam M. Sawye S. Fauquet C. M. (1999). Possible emergence of new geminiviruses by frequent recombination. *Virology* 265 218–225. 10.1006/viro.1999.0056 10600594

[B48] Posada D. Crandall K. A. (2001). Evaluation of methods for detecting recombination from DNA sequences: computer simulations. *Proc. Natl. Acad. Sci. U.S.A.* 98 13757–13762. 10.1073/pnas.241370698 11717435 PMC61114

[B49] Salunke B. K. Salunkhe R. C. Dhotre D. P. Walujkar S. A. Khandagale A. B. Chaudhari R. (2012). Determination of *Wolbachia* diversity in butterflies from Western Ghats, India, by a multigene approach. *Appl. Environ. Microbiol.* 78 4458–4467. 10.1128/AEM.07298-11 22504801 PMC3370507

[B50] Sanaei E. Charlat S. Engelstädter J. (2021). *Wolbachia* host shifts: routes, mechanisms, constraints and evolutionary consequences. *Biol. Rev.* 96 433–453. 10.1111/brv.12663 33128345

[B51] Shropshire J. D. Bordenstein S. R. (2019). Two-by-one model of cytoplasmic incompatibility: synthetic recapitulation by transgenic expression of cifA and cifB in *Drosophila*. *PLoS Genet.* 15:e1008221. 10.1371/journal.pgen.1008221 31242186 PMC6594578

[B52] Sintupachee S. Milne J. R. Poonchaisri S. Baimai V. Kittayapong P. (2006). Closely related *Wolbachia* strains within the pumpkin arthropod community and the potential for horizontal transmission via the plant. *Microb. Ecol.* 51 294–301. 10.1007/s00248-006-9036-x 16598632

[B53] Smith J. M. (1992). Analyzing the mosaic structure of genes. *J. Mol. Evol.* 34 126–129. 10.1007/BF00182389 1556748

[B54] Su C. Y. Zhu D. H. Yang X. H. (2021). Design and testing of effective primers for amplification of the orf7 gene of phage WO associated with *Andricus hakonensis*. *Insects* 12:713. 10.3390/insects12080713 34442279 PMC8397071

[B55] Swofford D. L. (2003). *PAUP*. Phylogenetic Analysis Using Parsimony (*and Other Methods), Version 4.* Sunderland, MA: Sinauer Associates.

[B56] Tagami Y. Miura K. (2004). Distribution and prevalence of *Wolbachia* in Japanese populations of Lepidoptera. *Insect Mol. Biol.* 13 359–364. 10.1111/j.0962-1075.2004.00492.x 15271207

[B57] Tanaka K. Furukawa S. Nikoh N. Sasaki T. Fukatsu T. (2009). Complete WO phage sequences reveal their dynamic evolutionary trajectories and putative functional elements required for integrationin to the *Wolbachia* genome. *Appl. Environ. Microbiol.* 75 5676–5686. 10.1128/aem.01172-09 19592535 PMC2737910

[B58] van Nieukerken E. J. Kaila L. Kitching I. J. Kristensen N. P. Lees D. C. Minet J. (2011). Order Lepidoptera Linnaeus, 1758. *Zootaxa* 3148 212–221. 10.11646/zootaxa.3148.1.41

[B59] Wang G. H. Sun B. F. Xiong T. L. Wang Y. K. Murfin K. E. Xiao J. H. (2016). Bacteriophage WO can mediate horizontal gene transfer in endosymbiotic *Wolbachia* genomes. *Front. Microbiol.* 7:1867. 10.3389/fmicb.2016.01867 27965627 PMC5126046

[B60] Wang M. Peng Y. Huang X. Jin Y. Peng Y. (2020). Incidence of *Wohlbachia*, *Cardinium*, *Spiroplasma* and phage WO in different geographical populations of *Chilo suppressalis* (Lepidoptera: Pyralidae) from China. *Entomol. News* 129 230–243. 10.3157/021.129.0302

[B61] Wang N. X. Jia S. S. Xu H. Liu Y. Huang D. W. (2016). Multiple horizontal transfers of bacteriophage WO and host *Wolbachia* in fig wasps in a closed community. *Front. Microbiol.* 7:136. 10.3389/fmicb.2016.00136 26913026 PMC4753557

[B62] Weinert L. A. Araujo-Jnr E. V. Ahmed M. Z. Welch J. J. (2015). The incidence of bacterial endosymbionts in terrestrial arthropods. *Proc. R. Soc. Lond. B* 282:20150249. 10.1098/rspb.2015.0249 25904667 PMC4424649

[B63] Werren J. H. Baldo L. Clark M. E. (2008). *Wolbachia*: master manipulators of invertebrate biology. *Nat. Rev. Microbiol.* 6 741–751. 10.1038/nrmicro1969 18794912

[B64] Werren J. H. Windsor D. Guo L. R. (1995). Distribution of *Wolbachia* among neotropical arthropods. *Proc. R. Soc. Lond. B* 262 197–204. 10.1098/rspb.1995.0196

[B65] Yang X. H. Zhu D. H. Liu Z. Zhao L. Su C. Y. (2013). High levels of multiple infections, recombination and horizontal transmission of *Wolbachia* in the *Andricus mukaigawae* (Hymenoptera; Cynipidae) communities. *PLoS One* 8:e78970. 10.1371/journal.pone.0078970 24250820 PMC3826730

[B66] Zhu D. H. Gao S. (2021). A prevalence survey of *Wolbachia* in butterflies from southern China. *Entomol. Exp. Appl.* 169 1157–1166. 10.1111/eea.13112

[B67] Zhu D. H. He Y. Y. Fan Y. S. Ma M. Y. Peng D. L. (2007). Negative evidence of parthenogenesis induction by *Wolbachia* in a gall wasp species, *Dryocosmus kuriphilus*. *Entomol. Exp. Appl.* 124 279–284. 10.1111/j.1570-7458.2007.00578.x

[B68] Zhu D. H. Su C. Y. Yang X. H. Abe Y. (2021). A case of intragenic recombination dramatically impacting the phage WO genetic diversity in gall wasps. *Front. Microbiol.* 12:694115. 10.3389/fmicb.2021.694115 34276627 PMC8279768

[B69] Zug R. Hammerstein P. (2012). Still a host of hosts for *Wolbachia*: analysis of recent data suggests that 40% of terrestrial arthropod species are infected. *PLoS One* 7:e38544. 10.1371/journal.pone.0038544 22685581 PMC3369835

