# Supplementary material for: High Levels of Multiple Phage WO Infections and Its Evolutionary Dynamics Associated With Wolbachia-Infected Butterflies
Source: Front Microbiol. 2022 Apr 21;13:865227. doi: 10.3389/fmicb.2022.865227 (PMC9070984; doi:10.3389/fmicb.2022.865227)
Supplement: Supplementary file 1 [file Table_1.DOCX]

Supplementary table 1 Sample information and *Wolbachia* infections in 57 butterfly species from China.

| Host species | | Location | Latitude/longitude | *Wolbachia* infection | Individuals screened |
| --- | --- | --- | --- | --- | --- |
| Family | Species |  |  |  |  |
| Hesperiidae | *Isoteinon* sp. | Zhangjiajie, Hunan | 29°20＇N/110°17＇E | + | 1 |
|  | *Notocrypta* sp. | Zhangjiajie, Hunan | 29°18＇N/110°30＇E | + | 1 |
|  | *Ochlodes thibetana* | Zhangjiajie, Hunan | 29°20＇N/110°17＇E | + | 1 |
| Lycaenidae | *Cupido lacturnus* | Chenzhou, Hunan | 26°4＇N/113°56＇E |  | 3 |
|  | *Curetis bulis* | Changsha, Hunan | 28°11＇N/112°56＇E |  | 1 |
|  | *Lampides boeticus* | Changsha, Hunan | 28°11＇N/112°56＇E |  | 2 |
|  | *Pseudozizeeria maha* | Changsha, Hunan | 28°11＇N/112°56＇E | + | 1 |
|  |  | Chenzhou, Hunan | 26°4＇N/113°56＇E | + | 1 |
| Nymphalidae | *Argyreus hyperbius* | Changsha, Hunan | 28°6＇N/113°1＇E |  | 1 |
|  |  | Shaoyang, Hunan | 27°21＇N/111°38＇E |  | 3 |
|  |  | Xiangtan, Hunan | 27°58＇N/113°0＇E |  | 2 |
|  | *Ariadne ariadne* | Xiangtan, Hunan | 27°58＇N/113°0＇E | + | 1 |
|  |  | Zhanjiang, Guangdong | 21°22＇N/110°16＇E | + | 1 |
|  | *Brenthis ino* ^1^ | Dandong, Liaoning | 40°14＇N/124°20＇E |  | 4 |
|  | *Charaxes bernardus* | Xiangtan, Hunan | 27°57＇N/113°0＇E |  | 1 |
|  | *Euploea core* | Zhanjiang, Guangdong | 21°22＇N/110°16＇E |  | 2 |
|  | *Euploea midamus* | Zhanjiang, Guangdong | 21°22＇N/110°16＇E |  | 2 |
|  | *Euploea tulliolus* | Zhanjiang, Guangdong | 21°22＇N/110°16＇E |  | 2 |
|  | *Euthalia pratti* | Jishou, Hunan | 28°20＇N/109°35＇E |  | 1 |
|  | *Fabriciana adippe* ^1^ | Dandong, Liaoning | 40°14＇N/124°20＇E |  | 7 |
|  | *Hestina persimilis* | Xiangtan, Hunan | 27°57＇N/113°0＇E |  | 2 |
|  | *Hypolimnas missipus* | Zhanjiang,Guangdong | 21°22＇N/110°16＇E |  | 1 |
|  | *Junonia almana* | Xiangtan, Hunan | 27°57＇N/113°0＇E | + | 2 |
|  | *Lethe confusa* | Jishou, Hunan | 28°20＇N/109°35＇E |  | 2 |
|  |  | Zhangjiajie, Hunan | 29°20＇N/110°17＇E |  | 1 |
|  | *Lethe syrcis* | Zhangjiajie, Hunan | 29°18＇N/110°30＇E |  | 1 |
|  | *Limenitis doerriesi* ^1^ | Dandong, Liaoning | 40°14＇N/124°20＇E | + | 4 |
|  | *Limenitis helmanni* | Shaoyang, Hunan | 27°21＇N/111°38＇E |  | 1 |
|  | *Limenitis sulpitia* | Jishou, Hunan | 28°20＇N/109°35＇E |  | 1 |
|  | *Mycalesis francisca* | Jishou, Hunan | 28°20＇N/109°35＇E | + | 1 |
|  | *Neope christi* | Jishou, Hunan | 28°20＇N/109°35＇E |  | 1 |
|  | *Nephargynnis anadyomene* ^1^ | Dandong, Liaoning | 40°14＇N/124°20＇E |  | 1 |
|  | *Neptis alwina* | Chenzhou, Hunan | 26°4＇N/113°56＇E |  | 1 |
|  | *Neptis hylas* | Xiangtan, Hunan | 27°58＇N/113°0＇E |  | 1 |
|  |  | Zhanjiang, Guangdong | 21°22＇N/110°16＇E |  | 3 |
|  | *Neptis sappho* | Changsha, Hunan | 28°11＇N/112°56＇E |  | 1 |
|  |  | Chenzhou, Hunan | 26°4＇N/113°56＇E |  | 2 |
|  |  | Jishou, Hunan | 28°20＇N/109°35＇E |  | 2 |
|  | *Polygonia c-aureum* | Xiangtan, Hunan | 27°58＇N/113°0＇E | + | 2 |
|  | *Sasakia charonda* | Jishou, Hunan | 28°20＇N/109°35＇E |  | 1 |
|  | *Stichophthalma* sp. | Chenzhou, Hunan | 26°4＇N/113°56＇E | + | 1 |
|  | *Stibochiona nicea* | Zhangjiajie, Hunan | 29°18＇N/110°30＇E |  | 1 |
|  | *Vanessa indica* | Changsha, Hunan | 28°11＇N/112°56＇E | + | 1 |
|  | *Ypthima baldus* | Zhangjiajie, Hunan | 29°18＇N/110°30＇E |  | 1 |
|  | *Ypthima motschulskyi* | Chenzhou, Hunan | 26°4＇N/113°56＇E |  | 1 |
|  | *Ypthima praenubila* | Jishou, Hunan | 28°20＇N/109°35＇E | + | 1 |
|  | *Ypthima* sp. | Zhangjiajie, Hunan | 29°18＇N/110°30＇E | + | 1 |
| Papilionidae | *Graphium chironides* | Changsha, Hunan | 28°6＇N/113°1＇E |  | 1 |
|  |  | Chenzhou, Hunan | 26°4＇N/113°56＇E |  | 1 |
|  | *Graphium sarpedon* | Changsha, Hunan | 28°11＇N/112°56＇E |  | 2 |
|  |  | Xiangtan, Hunan | 27°57＇N/113°0＇E |  | 8 |
|  | *Pachliopta aristolochiae* | Changsha, Hunan | 27°57＇N/113°0＇E |  | 2 |
|  |  | Shaoyang, Hunan | 28°11＇N/112°56＇E |  | 2 |
|  |  | Xiangtan, Hunan | 27°21＇N/111°38＇E |  | 1 |
|  | *Papilio bianor* | Changsha, Hunan | 28°6＇N/113°1＇E |  | 1 |
|  |  | Shaoyang, Hunan | 27°21＇N/111°38＇E |  | 1 |
|  |  | Xiangtan, Hunan | 27°58＇N/113°0＇E |  | 1 |
|  | *Papilio okinawensis* | Zhangjiajie, Hunan | 29°18＇N/110°30＇E |  | 1 |
|  | *Papilio polytes* | Changsha, Hunan | 28°8＇N/112°59＇E |  | 1 |
|  |  | Xiangtan, Hunan | 27°58＇N/113°0＇E |  | 2 |
|  |  | Zhanjiang, Guangdong | 21°22＇N/110°16＇E |  | 2 |
|  | *Papilio protenor* | Chenzhou, Hunan | 26°4＇N/113°56＇E |  | 1 |
|  | *Papilio xuthus* | Xiangtan, Hunan | 27°58＇N/113°0＇E |  | 1 |
|  | *Parnassius stubbendorfii* ^1^ | Dandong, Liaoning | 40°14＇N/124°20＇E | + | 10 |
| Pieridae | *Colias croceus* | Changsha, Hunan | 28°6＇N/113°1＇E | + | 1 |
|  |  | Jishou, Hunan | 28°20＇N/109°35＇E |  | 1 |
|  | *Delias acalis* | Jieyang, Guangdong | 23°15＇N/116°9＇E |  | 1 |
|  | *Delias agostina* | Chenzhou, Hunan | 26°4＇N/113°56＇E | + | 1 |
|  | *Eurema blanda* | Changsha, Hunan | 28°10＇N/112°53＇E | + | 2 |
|  |  | Xiangtan, Hunan | 27°58＇N/113°0＇E |  | 2 |
|  |  | Zhanjiang, Guangdong | 21°22＇N/110°16＇E |  | 1 |
|  |  | Zhangjiajie, Hunan | 29°18＇N/110°30＇E |  | 3 |
|  | *Eurema hecabe* | Changsha, Hunan | 28°10＇N/112°53＇E | + | 1 |
|  | *Leptosia nina* | Zhanjiang, Guangdong | 21°22＇N/110°16＇E | + | 2 |
|  | *Pieris canidia* | Changsha, Hunan | 28°8＇N/112°57＇E |  | 11 |
|  | *Pieris rapae* | Changsha, Hunan | 28°11＇N/112°58＇E |  | 7 |
|  |  | Xiangtan, Hunan | 27°58＇N/113°0＇E |  | 1 |

^1^ *Wolbachia* infection was detected in this study, and others by Zhu and Gao (2021).
