# Supplementary material for: High Levels of Multiple Phage WO Infections and Its Evolutionary Dynamics Associated With Wolbachia-Infected Butterflies
Source: Front Microbiol. 2022 Apr 21;13:865227. doi: 10.3389/fmicb.2022.865227 (PMC9070984; doi:10.3389/fmicb.2022.865227)
Supplement: Supplementary file 2 [file Presentation_1.PPTX]

## Slide 1
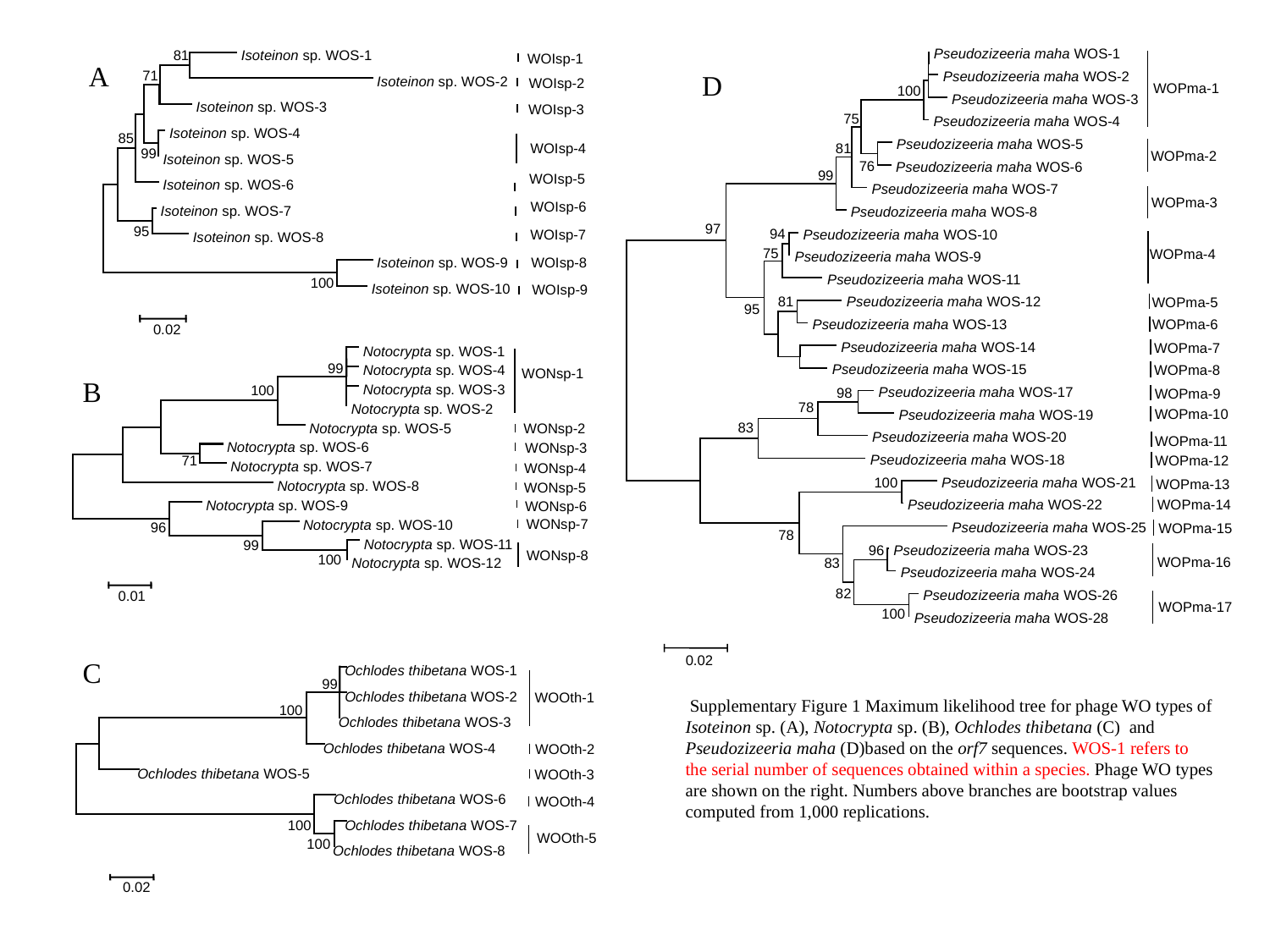

Pseudozizeeria maha WOS-1
 Pseudozizeeria maha WOS-2
100
 Pseudozizeeria maha WOS-3
75
 Pseudozizeeria maha WOS-4
 Pseudozizeeria maha WOS-5
81
76
 Pseudozizeeria maha WOS-6
99
 Pseudozizeeria maha WOS-7
 Pseudozizeeria maha WOS-8
97
94
 Pseudozizeeria maha WOS-10
75
 Pseudozizeeria maha WOS-9
 Pseudozizeeria maha WOS-11
81
 Pseudozizeeria maha WOS-12
95
 Pseudozizeeria maha WOS-13
 Pseudozizeeria maha WOS-14
 Pseudozizeeria maha WOS-15
 Pseudozizeeria maha WOS-17
98
78
 Pseudozizeeria maha WOS-19
83
 Pseudozizeeria maha WOS-20
 Pseudozizeeria maha WOS-18
100
 Pseudozizeeria maha WOS-21
 Pseudozizeeria maha WOS-22
 Pseudozizeeria maha WOS-25
78
96
 Pseudozizeeria maha WOS-23
83
 Pseudozizeeria maha WOS-24
82
 Pseudozizeeria maha WOS-26
100
 Pseudozizeeria maha WOS-28
0.02
WOPma-1
WOPma-2
WOPma-3
WOPma-4
WOPma-5
WOPma-6
WOPma-7
WOPma-8
WOPma-9
WOPma-10
WOPma-11
WOPma-12
WOPma-13
WOPma-14
WOPma-15
WOPma-16
WOPma-17
 Isoteinon sp. WOS-1
81
71
 Isoteinon sp. WOS-2
 Isoteinon sp. WOS-3
 Isoteinon sp. WOS-4
85
99
 Isoteinon sp. WOS-5
 Isoteinon sp. WOS-6
 Isoteinon sp. WOS-7
95
 Isoteinon sp. WOS-8
 Isoteinon sp. WOS-9
100
 Isoteinon sp. WOS-10
0.02
WOIsp-1
WOIsp-2
WOIsp-3
WOIsp-4
WOIsp-5
WOIsp-6
WOIsp-7
WOIsp-8
WOIsp-9
A
D
 Notocrypta sp. WOS-1
99
 Notocrypta sp. WOS-4
 Notocrypta sp. WOS-3
100
 Notocrypta sp. WOS-2
 Notocrypta sp. WOS-5
 Notocrypta sp. WOS-6
71
 Notocrypta sp. WOS-7
 Notocrypta sp. WOS-8
 Notocrypta sp. WOS-9
 Notocrypta sp. WOS-10
96
 Notocrypta sp. WOS-11
99
100
 Notocrypta sp. WOS-12
0.01
WONsp-1
WONsp-2
WONsp-3
WONsp-4
WONsp-5
WONsp-6
WONsp-7
WONsp-8
B
 Ochlodes thibetana WOS-1
99
 Ochlodes thibetana WOS-2
100
 Ochlodes thibetana WOS-3
 Ochlodes thibetana WOS-4
 Ochlodes thibetana WOS-5
 Ochlodes thibetana WOS-6
 Ochlodes thibetana WOS-7
100
100
 Ochlodes thibetana WOS-8
0.02
WOOth-1
WOOth-2
WOOth-3
WOOth-4
WOOth-5
C
 Supplementary Figure 1 Maximum likelihood tree for phage WO types of Isoteinon sp. (A), Notocrypta sp. (B), Ochlodes thibetana (C) and Pseudozizeeria maha (D)based on the orf7 sequences. WOS-1 refers to the serial number of sequences obtained within a species. Phage WO types are shown on the right. Numbers above branches are bootstrap values computed from 1,000 replications.

## Slide 2
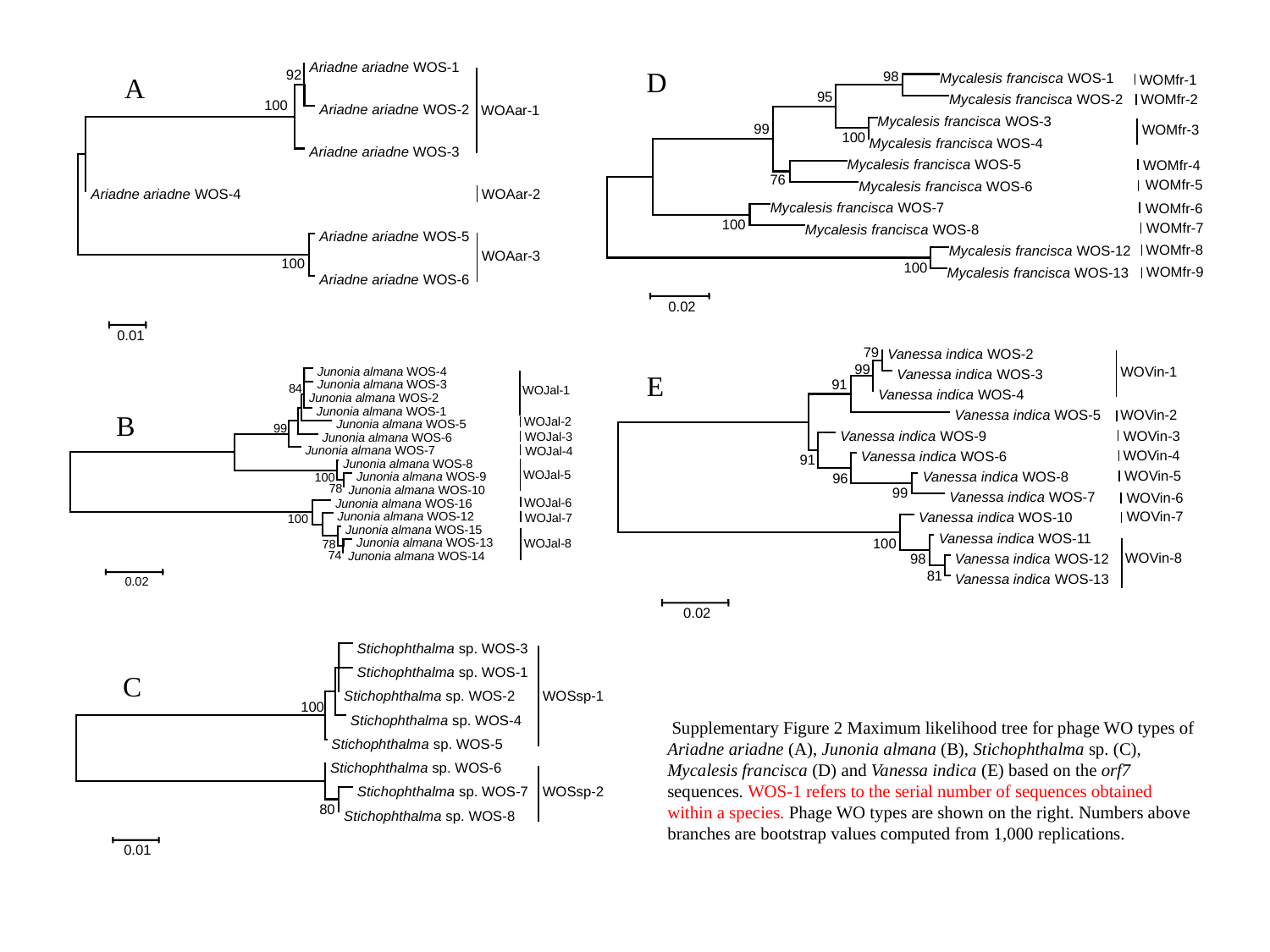

Ariadne ariadne WOS-1
92
100
 Ariadne ariadne WOS-2
 Ariadne ariadne WOS-3
 Ariadne ariadne WOS-4
 Ariadne ariadne WOS-5
100
 Ariadne ariadne WOS-6
0.01
WOAar-1
WOAar-2
WOAar-3
98
 Mycalesis francisca WOS-1
95
 Mycalesis francisca WOS-2
 Mycalesis francisca WOS-3
99
100
 Mycalesis francisca WOS-4
 Mycalesis francisca WOS-5
76
 Mycalesis francisca WOS-6
 Mycalesis francisca WOS-7
100
 Mycalesis francisca WOS-8
 Mycalesis francisca WOS-12
100
 Mycalesis francisca WOS-13
0.02
WOMfr-1
WOMfr-2
WOMfr-3
WOMfr-4
WOMfr-5
WOMfr-6
WOMfr-7
WOMfr-8
WOMfr-9
D
A
79
 Vanessa indica WOS-2
99
 Vanessa indica WOS-3
91
 Vanessa indica WOS-4
 Vanessa indica WOS-5
 Vanessa indica WOS-9
 Vanessa indica WOS-6
91
 Vanessa indica WOS-8
96
99
 Vanessa indica WOS-7
 Vanessa indica WOS-10
 Vanessa indica WOS-11
100
 Vanessa indica WOS-12
98
81
 Vanessa indica WOS-13
0.02
WOVin-1
WOVin-2
WOVin-3
WOVin-4
WOVin-5
WOVin-6
WOVin-7
WOVin-8
 Junonia almana WOS-4
 Junonia almana WOS-3
84
 Junonia almana WOS-2
 Junonia almana WOS-1
 Junonia almana WOS-5
99
 Junonia almana WOS-6
 Junonia almana WOS-7
 Junonia almana WOS-8
 Junonia almana WOS-9
100
78
 Junonia almana WOS-10
 Junonia almana WOS-16
 Junonia almana WOS-12
100
 Junonia almana WOS-15
 Junonia almana WOS-13
78
74
 Junonia almana WOS-14
0.02
WOJal-1
WOJal-2
WOJal-3
WOJal-4
WOJal-5
WOJal-6
WOJal-7
WOJal-8
E
B
 Stichophthalma sp. WOS-3
 Stichophthalma sp. WOS-1
 Stichophthalma sp. WOS-2
100
 Stichophthalma sp. WOS-4
 Stichophthalma sp. WOS-5
 Stichophthalma sp. WOS-6
 Stichophthalma sp. WOS-7
80
 Stichophthalma sp. WOS-8
0.01
WOSsp-1
WOSsp-2
C
 Supplementary Figure 2 Maximum likelihood tree for phage WO types of Ariadne ariadne (A), Junonia almana (B), Stichophthalma sp. (C), Mycalesis francisca (D) and Vanessa indica (E) based on the orf7 sequences. WOS-1 refers to the serial number of sequences obtained within a species. Phage WO types are shown on the right. Numbers above branches are bootstrap values computed from 1,000 replications.

## Slide 3
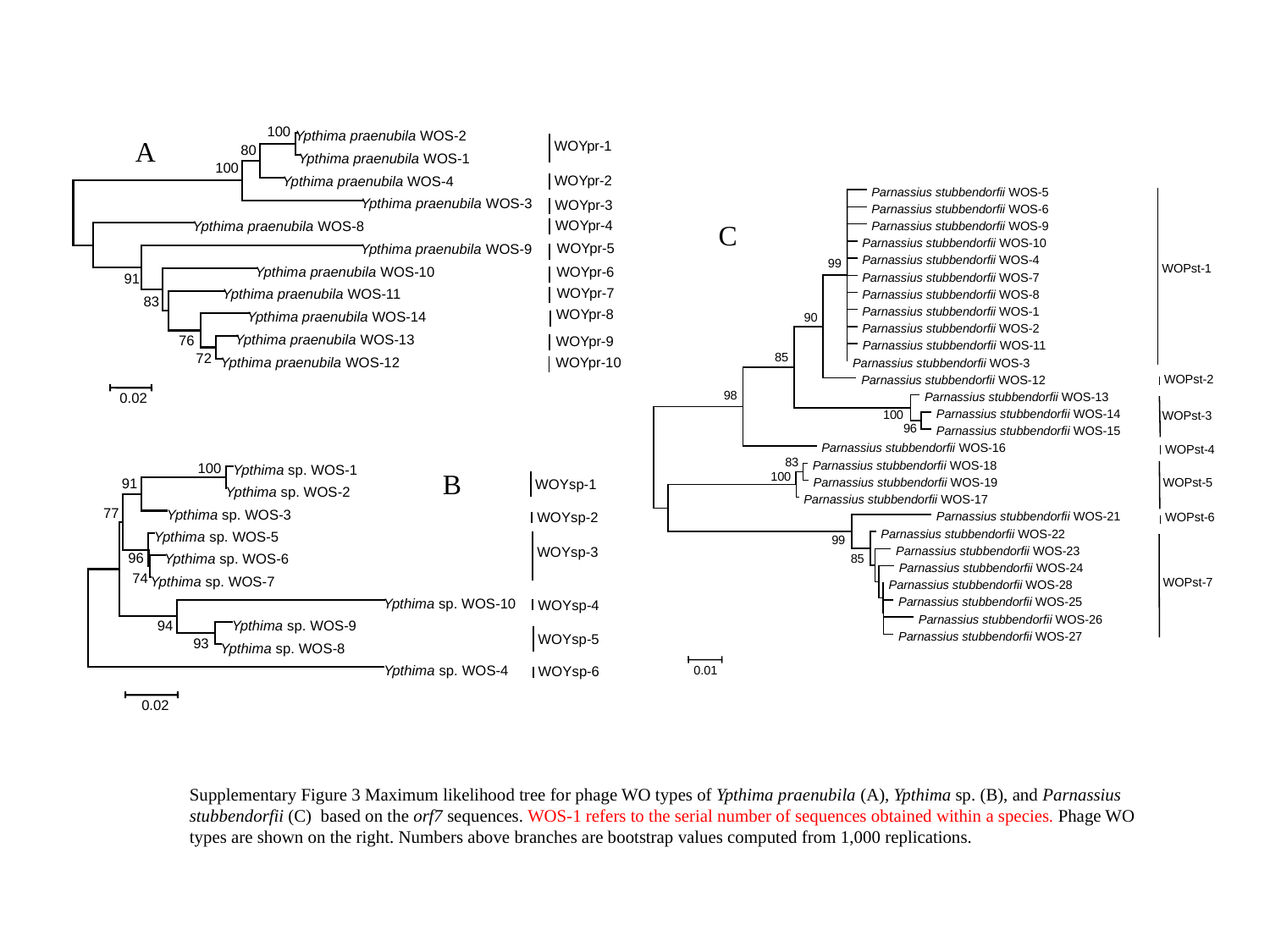

100
 Ypthima praenubila WOS-2
80
 Ypthima praenubila WOS-1
100
 Ypthima praenubila WOS-4
 Ypthima praenubila WOS-3
 Ypthima praenubila WOS-8
 Ypthima praenubila WOS-9
 Ypthima praenubila WOS-10
91
 Ypthima praenubila WOS-11
83
 Ypthima praenubila WOS-14
 Ypthima praenubila WOS-13
76
72
 Ypthima praenubila WOS-12
0.02
WOYpr-1
WOYpr-2
WOYpr-3
WOYpr-4
WOYpr-5
WOYpr-6
WOYpr-7
WOYpr-8
WOYpr-9
WOYpr-10
A
 Parnassius stubbendorfii WOS-5
 Parnassius stubbendorfii WOS-6
 Parnassius stubbendorfii WOS-9
 Parnassius stubbendorfii WOS-10
 Parnassius stubbendorfii WOS-4
99
 Parnassius stubbendorfii WOS-7
 Parnassius stubbendorfii WOS-8
 Parnassius stubbendorfii WOS-1
90
 Parnassius stubbendorfii WOS-2
 Parnassius stubbendorfii WOS-11
85
 Parnassius stubbendorfii WOS-3
 Parnassius stubbendorfii WOS-12
98
 Parnassius stubbendorfii WOS-13
 Parnassius stubbendorfii WOS-14
100
96
 Parnassius stubbendorfii WOS-15
 Parnassius stubbendorfii WOS-16
83
 Parnassius stubbendorfii WOS-18
100
 Parnassius stubbendorfii WOS-19
 Parnassius stubbendorfii WOS-17
 Parnassius stubbendorfii WOS-21
 Parnassius stubbendorfii WOS-22
99
 Parnassius stubbendorfii WOS-23
85
 Parnassius stubbendorfii WOS-24
 Parnassius stubbendorfii WOS-28
 Parnassius stubbendorfii WOS-25
 Parnassius stubbendorfii WOS-26
 Parnassius stubbendorfii WOS-27
0.01
WOPst-1
WOPst-2
WOPst-3
WOPst-4
WOPst-5
WOPst-6
WOPst-7
C
100
 Ypthima sp. WOS-1
91
 Ypthima sp. WOS-2
77
 Ypthima sp. WOS-3
 Ypthima sp. WOS-5
96
 Ypthima sp. WOS-6
74
 Ypthima sp. WOS-7
 Ypthima sp. WOS-10
94
 Ypthima sp. WOS-9
93
 Ypthima sp. WOS-8
 Ypthima sp. WOS-4
0.02
WOYsp-1
WOYsp-2
WOYsp-3
WOYsp-4
WOYsp-5
WOYsp-6
B
Supplementary Figure 3 Maximum likelihood tree for phage WO types of Ypthima praenubila (A), Ypthima sp. (B), and Parnassius stubbendorfii (C) based on the orf7 sequences. WOS-1 refers to the serial number of sequences obtained within a species. Phage WO types are shown on the right. Numbers above branches are bootstrap values computed from 1,000 replications.

## Slide 4
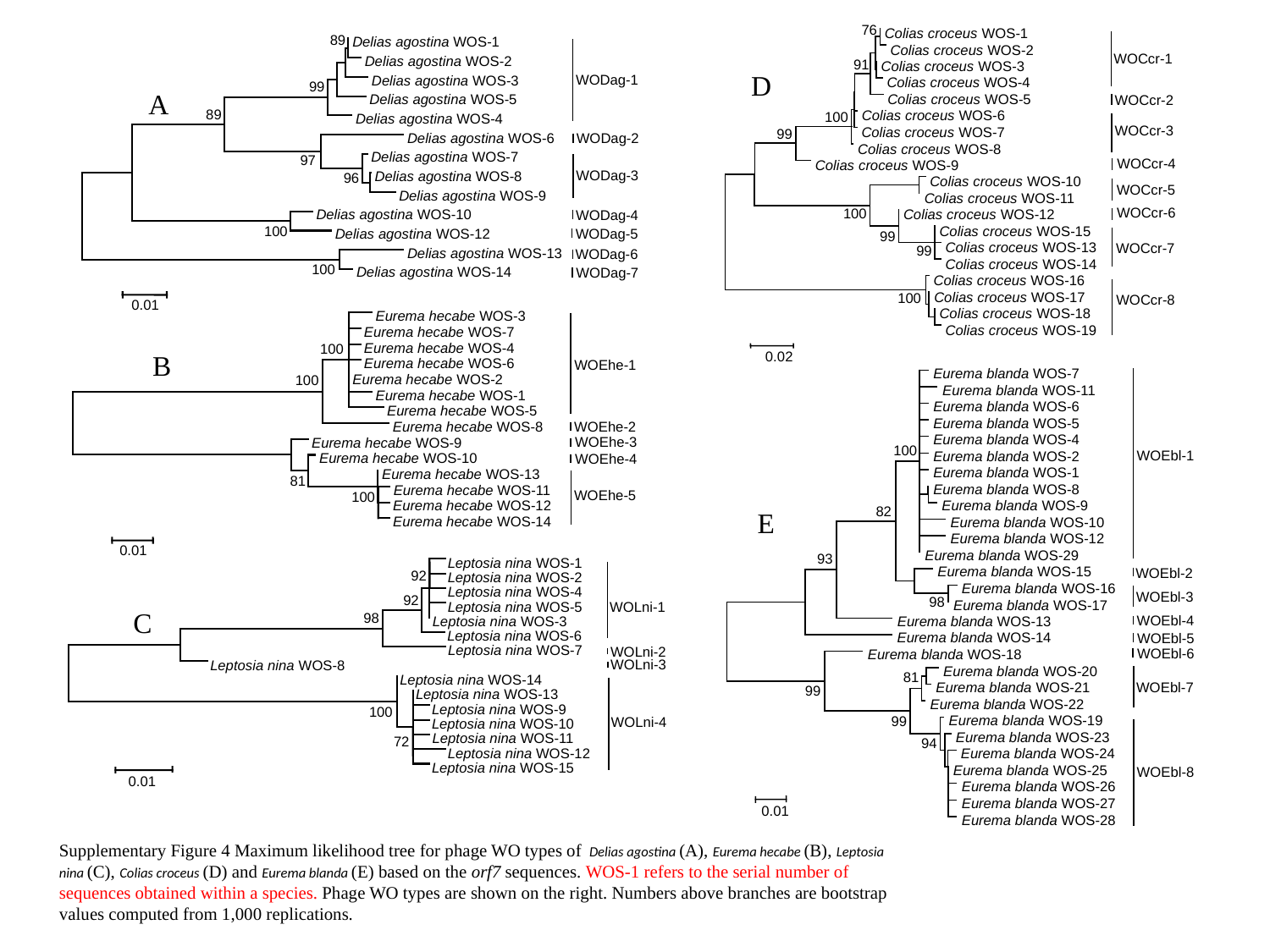

76
 Colias croceus WOS-1
 Colias croceus WOS-2
91
 Colias croceus WOS-3
 Colias croceus WOS-4
 Colias croceus WOS-5
 Colias croceus WOS-6
100
 Colias croceus WOS-7
99
 Colias croceus WOS-8
 Colias croceus WOS-9
 Colias croceus WOS-10
 Colias croceus WOS-11
100
 Colias croceus WOS-12
 Colias croceus WOS-15
99
 Colias croceus WOS-13
99
 Colias croceus WOS-14
 Colias croceus WOS-16
 Colias croceus WOS-17
100
 Colias croceus WOS-18
 Colias croceus WOS-19
0.02
WOCcr-1
WOCcr-2
WOCcr-3
WOCcr-4
WOCcr-5
WOCcr-6
WOCcr-7
WOCcr-8
89
 Delias agostina WOS-1
 Delias agostina WOS-2
 Delias agostina WOS-3
99
 Delias agostina WOS-5
89
 Delias agostina WOS-4
 Delias agostina WOS-6
 Delias agostina WOS-7
97
 Delias agostina WOS-8
96
 Delias agostina WOS-9
 Delias agostina WOS-10
100
 Delias agostina WOS-12
 Delias agostina WOS-13
100
 Delias agostina WOS-14
0.01
WODag-1
WODag-2
WODag-3
WODag-4
WODag-5
WODag-6
WODag-7
D
A
 Eurema hecabe WOS-3
 Eurema hecabe WOS-7
 Eurema hecabe WOS-4
100
 Eurema hecabe WOS-6
 Eurema hecabe WOS-2
100
 Eurema hecabe WOS-1
 Eurema hecabe WOS-5
 Eurema hecabe WOS-8
 Eurema hecabe WOS-9
 Eurema hecabe WOS-10
 Eurema hecabe WOS-13
81
 Eurema hecabe WOS-11
100
 Eurema hecabe WOS-12
 Eurema hecabe WOS-14
0.01
WOEhe-1
WOEhe-2
WOEhe-3
WOEhe-4
WOEhe-5
B
 Eurema blanda WOS-7
 Eurema blanda WOS-11
 Eurema blanda WOS-6
 Eurema blanda WOS-5
 Eurema blanda WOS-4
100
 Eurema blanda WOS-2
 Eurema blanda WOS-1
 Eurema blanda WOS-8
 Eurema blanda WOS-9
82
 Eurema blanda WOS-10
 Eurema blanda WOS-12
 Eurema blanda WOS-29
93
 Eurema blanda WOS-15
 Eurema blanda WOS-16
98
 Eurema blanda WOS-17
 Eurema blanda WOS-13
 Eurema blanda WOS-14
 Eurema blanda WOS-18
 Eurema blanda WOS-20
81
 Eurema blanda WOS-21
99
 Eurema blanda WOS-22
 Eurema blanda WOS-19
99
 Eurema blanda WOS-23
94
 Eurema blanda WOS-24
 Eurema blanda WOS-25
 Eurema blanda WOS-26
 Eurema blanda WOS-27
0.01
 Eurema blanda WOS-28
WOEbl-1
WOEbl-2
WOEbl-3
WOEbl-4
WOEbl-5
WOEbl-6
WOEbl-7
WOEbl-8
E
 Leptosia nina WOS-1
92
 Leptosia nina WOS-2
 Leptosia nina WOS-4
92
 Leptosia nina WOS-5
98
 Leptosia nina WOS-3
 Leptosia nina WOS-6
 Leptosia nina WOS-7
 Leptosia nina WOS-8
 Leptosia nina WOS-14
 Leptosia nina WOS-13
 Leptosia nina WOS-9
100
 Leptosia nina WOS-10
 Leptosia nina WOS-11
72
 Leptosia nina WOS-12
 Leptosia nina WOS-15
0.01
WOLni-1
WOLni-2
WOLni-3
WOLni-4
C
Supplementary Figure 4 Maximum likelihood tree for phage WO types of Delias agostina (A), Eurema hecabe (B), Leptosia nina (C), Colias croceus (D) and Eurema blanda (E) based on the orf7 sequences. WOS-1 refers to the serial number of sequences obtained within a species. Phage WO types are shown on the right. Numbers above branches are bootstrap values computed from 1,000 replications.
