# Supplementary material for: High Levels of Multiple Phage WO Infections and Its Evolutionary Dynamics Associated With Wolbachia-Infected Butterflies
Source: Front Microbiol. 2022 Apr 21;13:865227. doi: 10.3389/fmicb.2022.865227 (PMC9070984; doi:10.3389/fmicb.2022.865227)
Supplement: Supplementary file 3 [file Presentation_2.PPTX]

## Slide 1
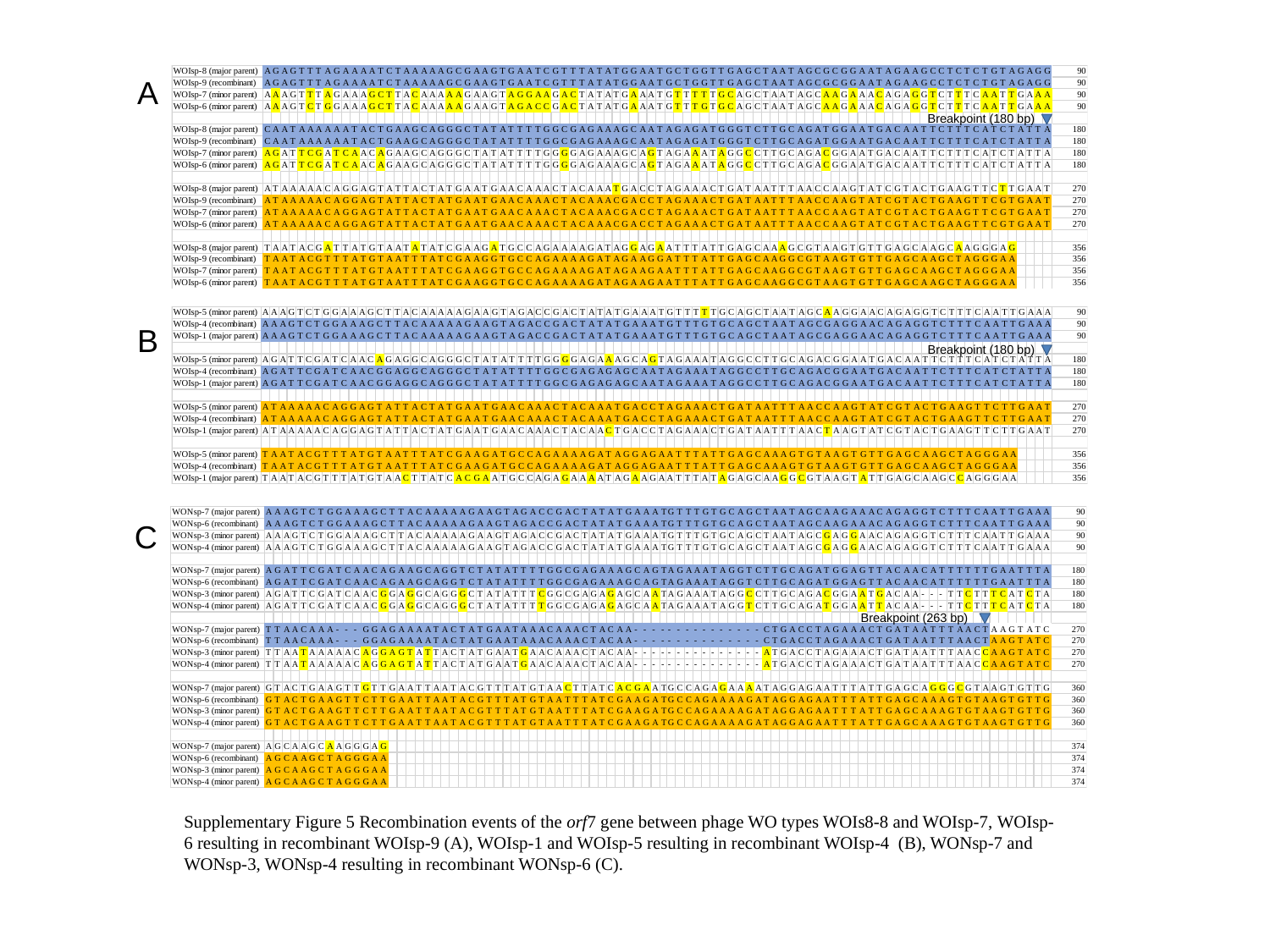

A
Breakpoint (180 bp)
B
Breakpoint (180 bp)
C
Breakpoint (263 bp)
Supplementary Figure 5 Recombination events of the orf7 gene between phage WO types WOIs8-8 and WOIsp-7, WOIsp-6 resulting in recombinant WOIsp-9 (A), WOIsp-1 and WOIsp-5 resulting in recombinant WOIsp-4 (B), WONsp-7 and WONsp-3, WONsp-4 resulting in recombinant WONsp-6 (C).

## Slide 2
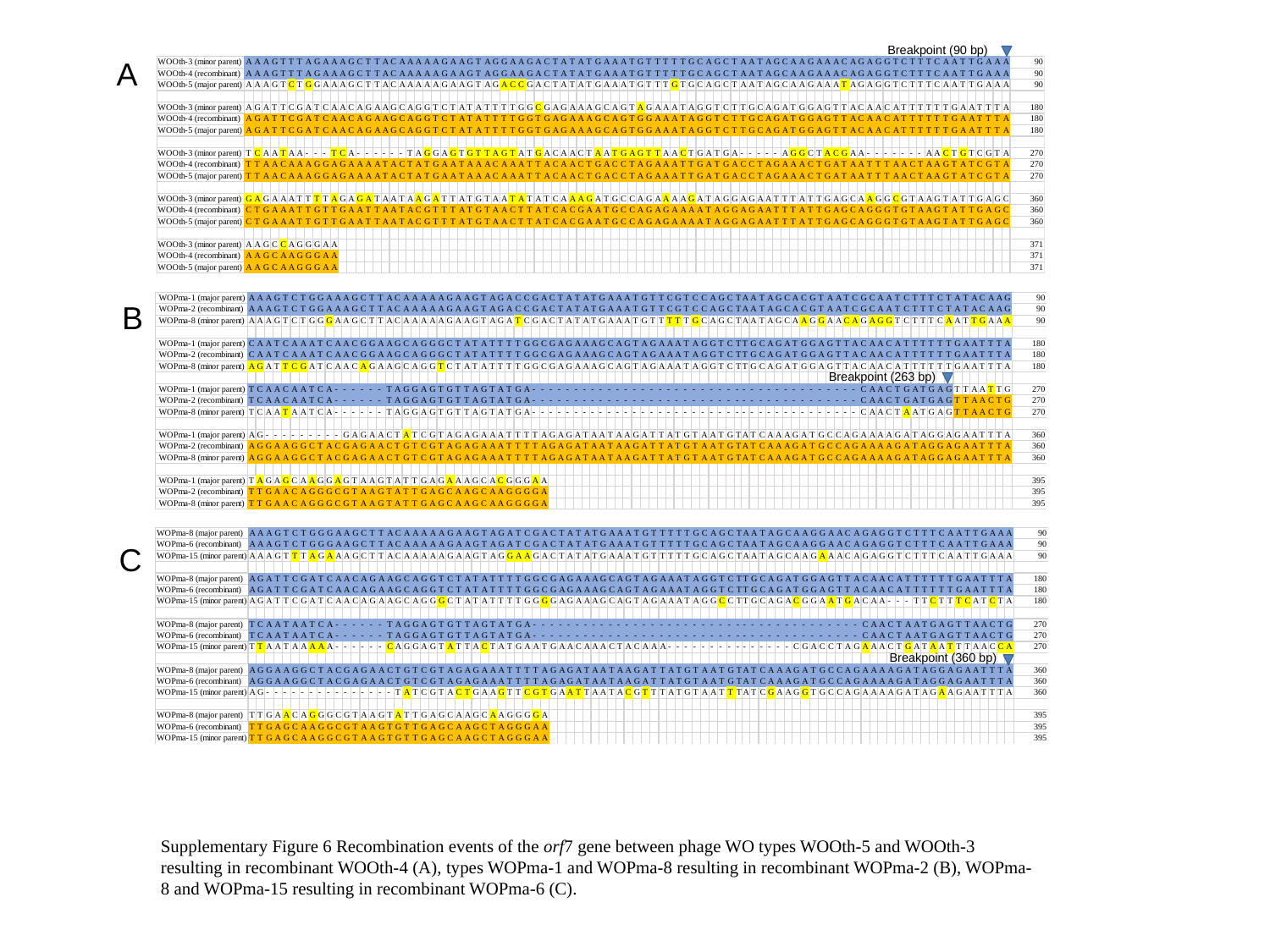

Breakpoint (90 bp)
A
B
Breakpoint (263 bp)
C
Breakpoint (360 bp)
Supplementary Figure 6 Recombination events of the orf7 gene between phage WO types WOOth-5 and WOOth-3 resulting in recombinant WOOth-4 (A), types WOPma-1 and WOPma-8 resulting in recombinant WOPma-2 (B), WOPma-8 and WOPma-15 resulting in recombinant WOPma-6 (C).

## Slide 3
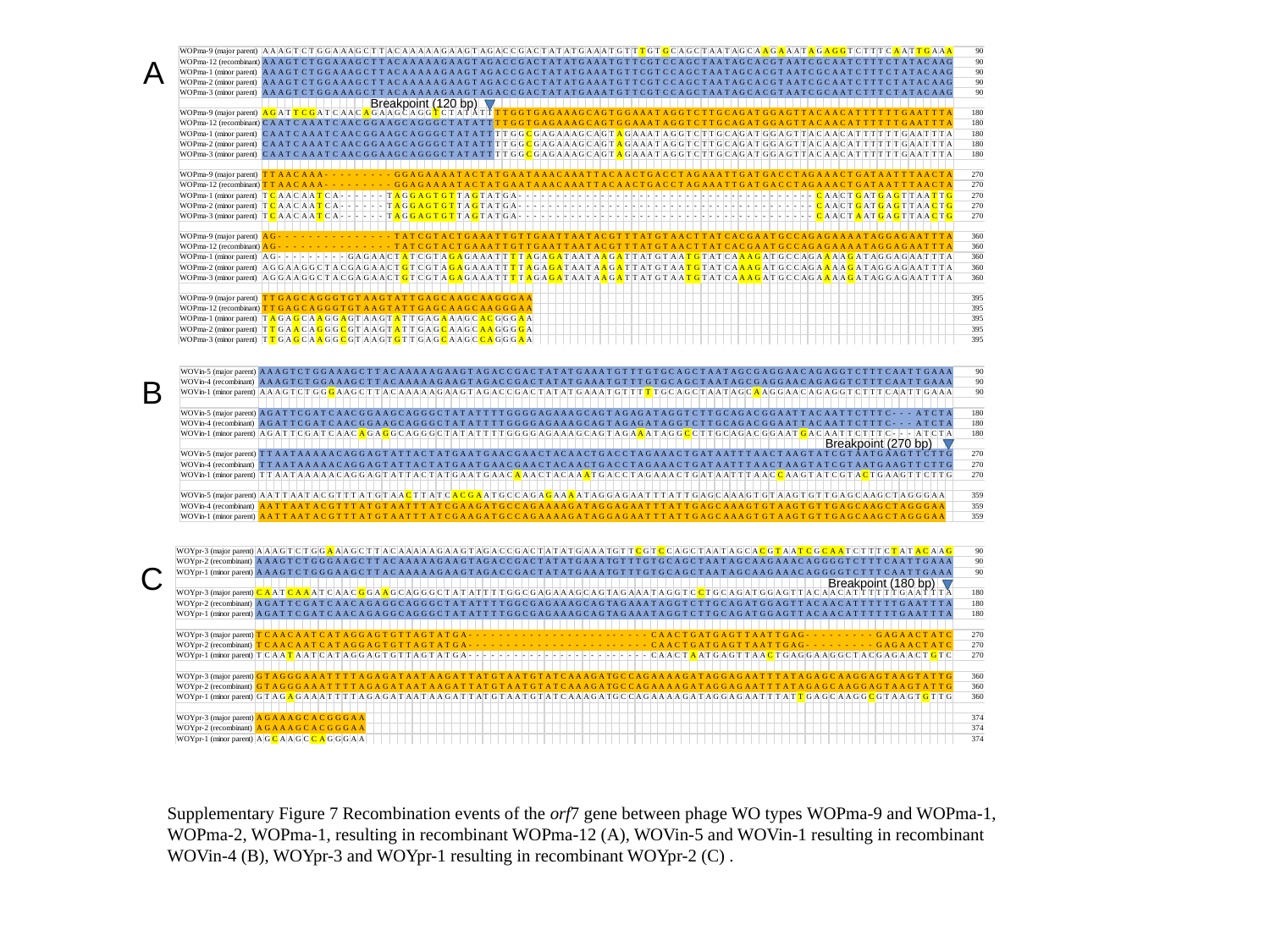

A
Breakpoint (120 bp)
B
Breakpoint (270 bp)
C
Breakpoint (180 bp)
Supplementary Figure 7 Recombination events of the orf7 gene between phage WO types WOPma-9 and WOPma-1, WOPma-2, WOPma-1, resulting in recombinant WOPma-12 (A), WOVin-5 and WOVin-1 resulting in recombinant WOVin-4 (B), WOYpr-3 and WOYpr-1 resulting in recombinant WOYpr-2 (C) .

## Slide 4
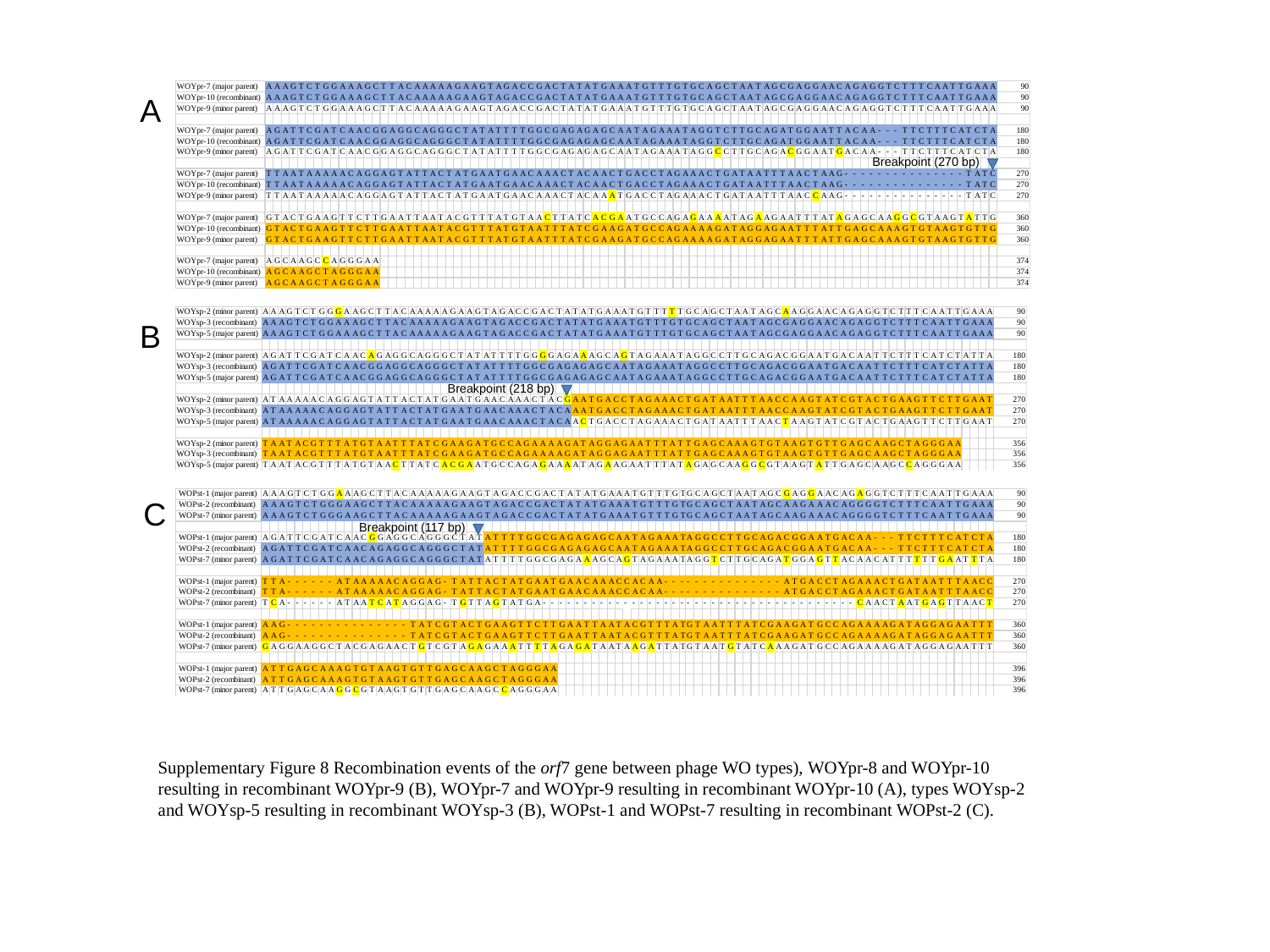

A
Breakpoint (270 bp)
B
Breakpoint (218 bp)
C
Breakpoint (117 bp)
Supplementary Figure 8 Recombination events of the orf7 gene between phage WO types), WOYpr-8 and WOYpr-10 resulting in recombinant WOYpr-9 (B), WOYpr-7 and WOYpr-9 resulting in recombinant WOYpr-10 (A), types WOYsp-2 and WOYsp-5 resulting in recombinant WOYsp-3 (B), WOPst-1 and WOPst-7 resulting in recombinant WOPst-2 (C).

## Slide 5
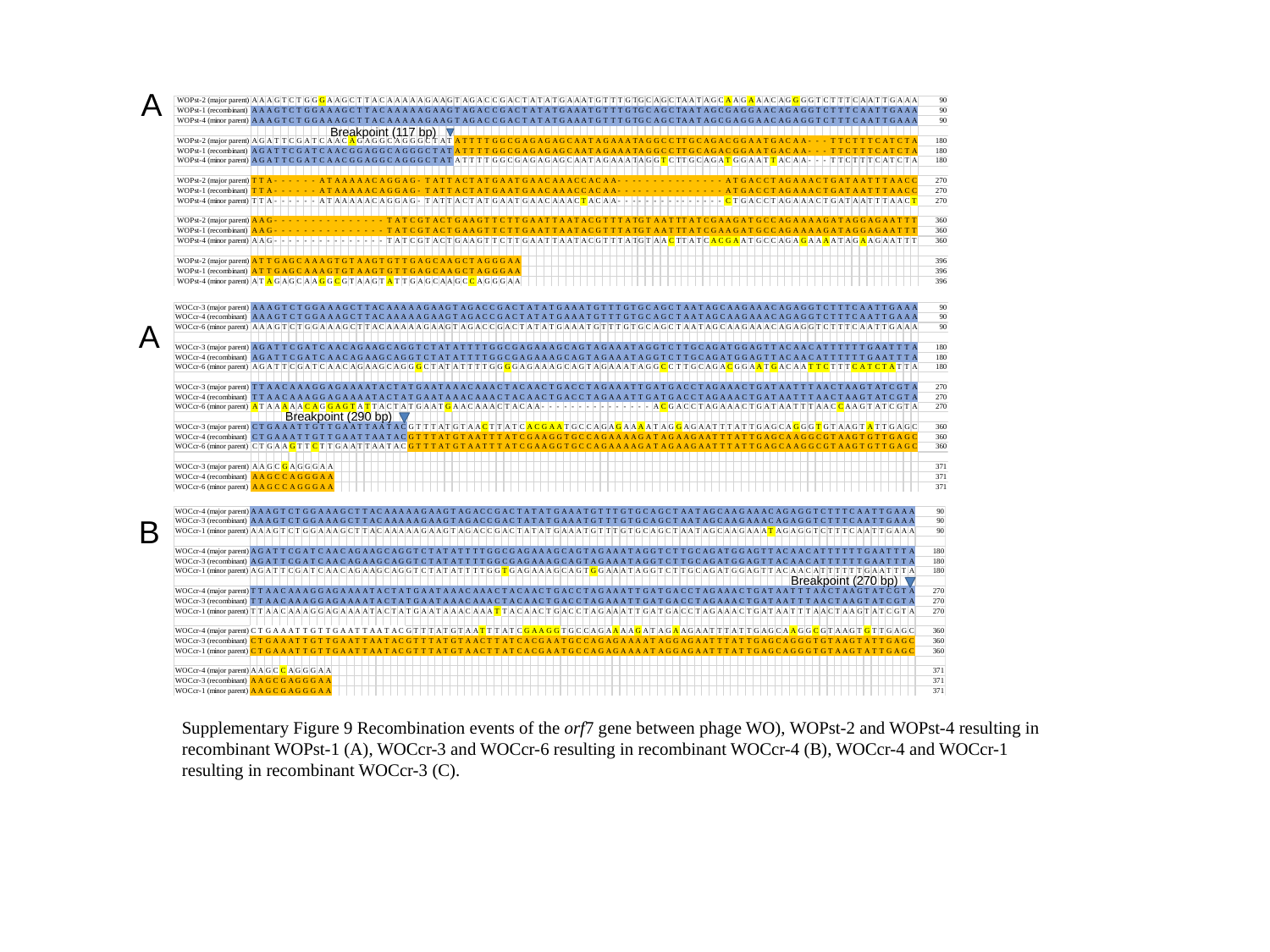

A
Breakpoint (117 bp)
A
Breakpoint (290 bp)
B
Breakpoint (270 bp)
Supplementary Figure 9 Recombination events of the orf7 gene between phage WO), WOPst-2 and WOPst-4 resulting in recombinant WOPst-1 (A), WOCcr-3 and WOCcr-6 resulting in recombinant WOCcr-4 (B), WOCcr-4 and WOCcr-1 resulting in recombinant WOCcr-3 (C).

## Slide 6
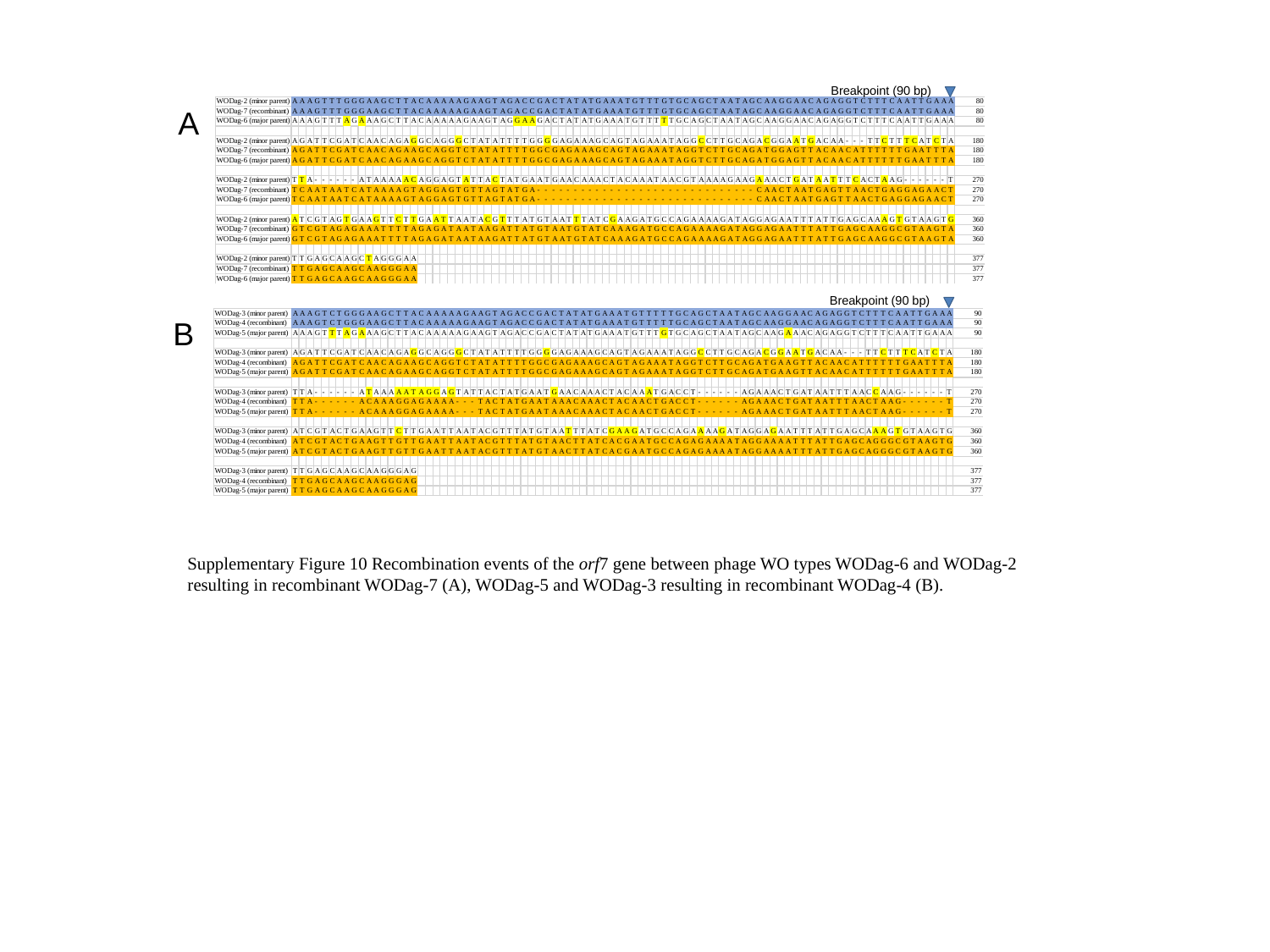

Breakpoint (90 bp)
A
Breakpoint (90 bp)
B
Supplementary Figure 10 Recombination events of the orf7 gene between phage WO types WODag-6 and WODag-2 resulting in recombinant WODag-7 (A), WODag-5 and WODag-3 resulting in recombinant WODag-4 (B).

## Slide 7
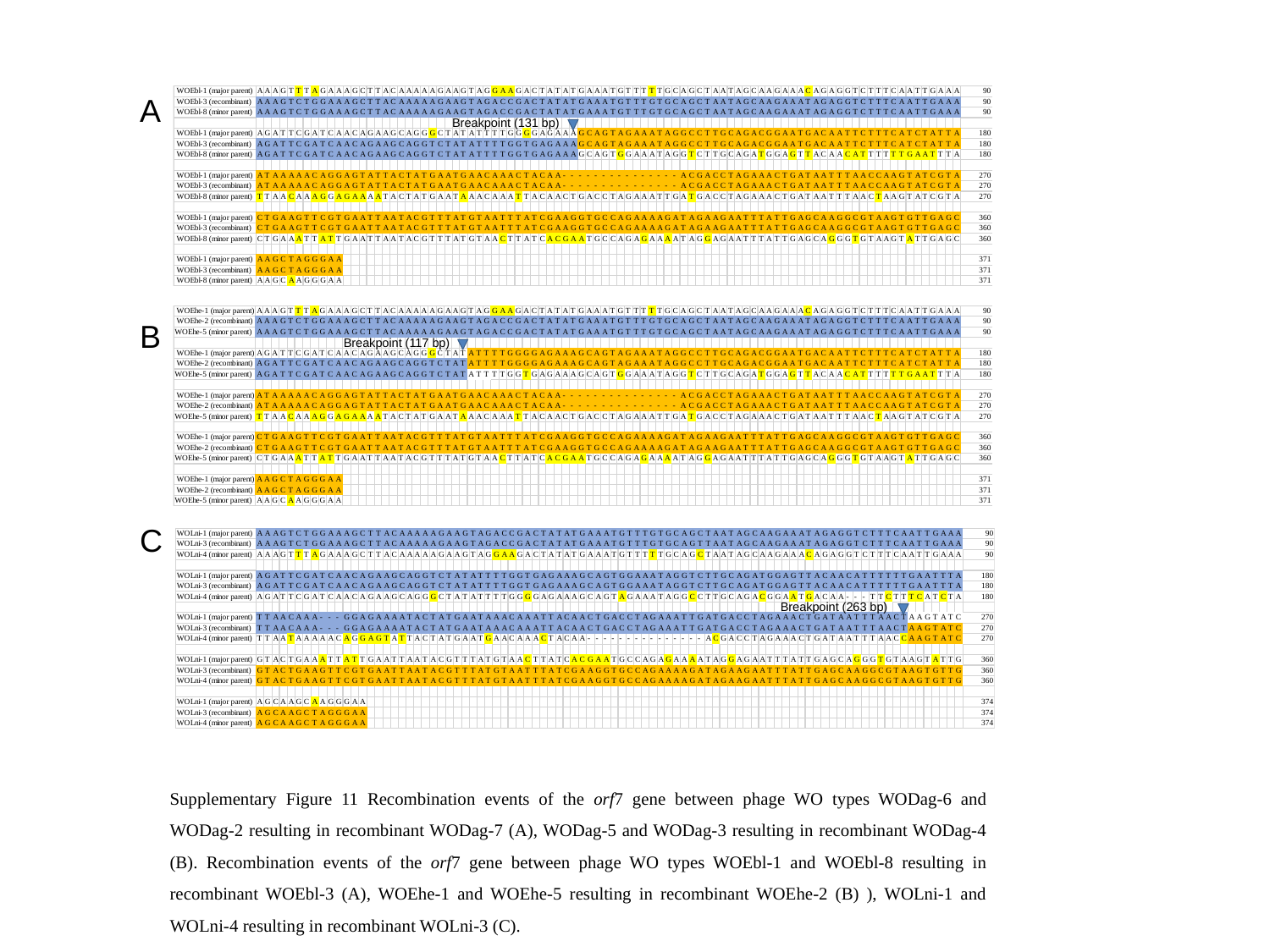

A
Breakpoint (131 bp)
B
Breakpoint (117 bp)
C
Breakpoint (263 bp)
Supplementary Figure 11 Recombination events of the orf7 gene between phage WO types WODag-6 and WODag-2 resulting in recombinant WODag-7 (A), WODag-5 and WODag-3 resulting in recombinant WODag-4 (B). Recombination events of the orf7 gene between phage WO types WOEbl-1 and WOEbl-8 resulting in recombinant WOEbl-3 (A), WOEhe-1 and WOEhe-5 resulting in recombinant WOEhe-2 (B) ), WOLni-1 and WOLni-4 resulting in recombinant WOLni-3 (C).
